# Supplementary material for: Mutational signature dynamics shaping the evolution of oesophageal adenocarcinoma
Source: Nat Commun. 2023 Jul 15;14:4239. doi: 10.1038/s41467-023-39957-6 (PMC10349863; doi:10.1038/s41467-023-39957-6)
Supplement: Supplementary file 1 — Supplementary Information [file 41467_2023_39957_MOESM1_ESM.pdf]

SUPPLEMENTARY MATERIAL

Mutational signature dynamics shaping the evolution of oesophageal adenocarcinoma

Sujath Abbas[1], Oriol Pich[2], Ginny Devonshire[3], Shawn Zamani[1], Annalise Katz-Summercorn[1], Sarah Killcoyne[1,4], Calvin Cheah[1], Barbara Nutzinger[1], Nicola Grehan[1], Nuria Lopez-Bigas[2,5,6], OCCAMS, Rebecca C. Fitzgerald[1,*], Maria Secrier[7,*]

[1]Early Cancer Institute, University of Cambridge, Cambridge, UK
[2]Institute for Research in Biomedicine (IRB Barcelona), The Barcelona Institute of Science and Technology, Barcelona, Spain
[3]Cancer Research UK Cambridge Institute, University of Cambridge, Cambridge, UK
[4]European Molecular Biology Laboratory, European Bioinformatics Institute (EMBL-EBI), Hinxton, UK
[5]Institució Catalana de Recerca i Estudis Avançats (ICREA), Barcelona, Spain
[6]Centro de Investigación Biomédica en Red en Cáncer (CIBERONC), Instituto de Salud Carlos III, Madrid, Spain
[7]UCL Genetics Institute, Department of Genetics, Evolution and Environment, University College London, London, UK

*co-corresponding

SUPPLEMENTARY TABLES

Supplementary Table 1. Clinical characteristics of the cohort.

Variable (unit)	Measure/Level	OAC(n=645)	Barrett Oesophagus (n=147)
**Age (years)**	(median,IQR)	67.1 (59.2-74.2)	**68 (62.0-75.9)**
**Gender**	Female	85 (13.2%)	**26 (17.7%)**
	Male	560 (86.8%)	**121 (82.3%)**
**Exposures**			
**Smoking status**		535 (82.9%)	**142 (96.6%)**
	Current	92 (14.2%)	**84 (57.1%)**
	Former	293 (45.4%)	**24 (16.3%)**
	Never	150 (23.2%)	**34 (23.1%)**
	Missing data	110 (17.0%)	**6 (4.1%)**
**Alcohol use(standard units/week)**	Mean(min-max)	6.2 (1-70)	**1.0 (1-3)**
**Proton pump inhibitor (PPI)/Acid suppressant use**		512 (79.4%)	**140 (95.2%)**
	Current Use	255 (39.5%)	**126 (85.7%)**
	Past Use	57 (8.8%)	**1 (0.7%)**
	Never	200 (31.0%)	**13 (8.8%)**
	Missing data	133 (20.6%)	**8 (5.4%)**
**Non-steroidal anti-inflammatory drug use**		322 (49.9%)	**116 (78.9%)**
**(NSAID)**	Current Use	128 (19.8%)	**37 (25.2%)**
	Past Use	51 (7.9%)	**3 (2.0%)**
	Never	143(22.1%)	**76 (51.7%)**
	Missing data	323 (50.0%)	**32 (21.8%)**
**Body mass index (BMI; kg/m^2)**	(median, IQR)	27.3 (24.4-31.2)	**29 (13.5-43.9)**
**Overall Survival (weeks)**	(median, IQR)	109 (57-188)	**Not Available**
**Barrett Oesophagus origin$^{\Psi}$**			
**Patient grade**	NDBO (NP)	**Not Applicable**	**27 (18.4%)**
	NDBO (PP)		**12 (8.2%)**
	LGD		**15 (10.2%)**
	HGD		**25 (17.0%)**
	IMC		**21 (14.3%)**
	Adjacent to tumour		**47 (32.0%)**
**Diagnosis**			
**Pre-treatment Tumour Stage**		511 (79.2%)	**Not Applicable**
	T0	1 (0.1%)	
	T1	30 (4.7%)	
	T2	83 (12.9%)	

	T3	353 (54.7%)	
	T4	25 (3.9%)	
	Tx (*in situ*)	19 (2.9%)	
	Missing data	134 (20.7%)	
**Pre-treatment nodal involvement (CT)**		571 (88.5%)	**Not Applicable**
	Positive	403 (62.5%)	
	Negative	168 (26.0%)	
	Missing data	74 (11.5%)	
**Pre-treatment distant metastases (CT)**		545 (84.5%)	
	Positive	39 (6.0%)	
	Negative	459 (71.2%)	
	Mx	47 (7.3%)	
	Missing data	100 (15.5%)	
**Pre-treatment Siewert Classification**		304 (47.13%)	
	Type I	143 (22.1%)	
	Type II	116 (18.0%)	
	Type III	45 (7.0%)	
	Missing data	341 (52.8%)	
**Therapy***			
**NeoAdj.Chemotherapy**			**Not Applicable**
**5FU**	Treated	57 (8.8%)	
	Not Treated	177 (27.4%)	
**Capecitabine**	Treated	276 (42.8%)	
	Not Treated	47 (7.3%)	
**Cisplatin**	Treated	272 (42.2%)	
	Not Treated	42 (6.5%)	
**Epirubicin**	Treated	271 (42.0%)	
	Not Treated	47 (7.3%)	
**Oxaliplatin**	Treated	49 (7.6%)	
	Not Treated	190 (29.4%)	
**Lapatinib**	Treated	5 (0.8%)	
	Not Treated	198 (30.7%)	
**Surgery**	Yes	452 (70.0%)	
	No	151 (23.4%)	

$^{\Psi}$ NDBO = non-dysplastic Barrett Oesophagus; NP = non-progressor; PP = pre-progressor; LGD = lower grade dysplasia; HGD = higher grade dysplasia; IMC = intramucosal carcinoma.
* Treatment was based on a planned protocol, so a combination of different drugs were administered.

Supplementary Table 2. Site of origin for metastatic samples.

Characteristic	N = 59[1]
Site	
Adrenal	2 (3.4%)
Brain	4 (6.8%)
Falciformligament	1 (1.7%)
Left colon (peritoneal)	1 (1.7%)
Left hemidiaphragm	1 (1.7%)
Left lung	1 (1.7%)
Liver	9 (15%)
Local lymph node	8 (14%)
Local lymph node (left gastric)	8 (14%)
Local lymph node (liver hilum)	1 (1.7%)
Local lymph node (mediastinal)	1 (1.7%)
Lymph node	1 (1.7%)
Neck lymph node	7 (12%)
Pancreas	2 (3.4%)
Para-aortic lymph node	5 (8.5%)
Pelvis (peritoneal)	1 (1.7%)
Right colon (peritoneal)	1 (1.7%)
Right colon (serosa)	1 (1.7%)
Right hemidiaphragm	1 (1.7%)
Right lung	1 (1.7%)
Right pleura	1 (1.7%)
Small bowel mesentery lymph node	1 (1.7%)

[1] n (%)

Supplementary Table 3. Number of samples with/without evidence of HRD signature exposure.

	Barretts	PrimaryTumour	Metastasis
yes	3	58	0
no	158	719	59

Supplementary Table 4. Number of samples with/without evidence of colibactin signature exposure (SBS41 and ID18).

	Barretts	PrimaryTumour	Metastasis
yes	5	10	3
no	156	767	56

Supplementary Table 5. Overall survival outcomes modelled on signature prevalence and clinical/demographic confounders. The results of a Cox proportional hazards model are shown (global log-rank p-value 2.2e-05). Only signature SBS30 was significantly prognostic after accounting for potential confounders. Hazard ratio estimates are presented as boxes, with lines indicating 95% confidence intervals.

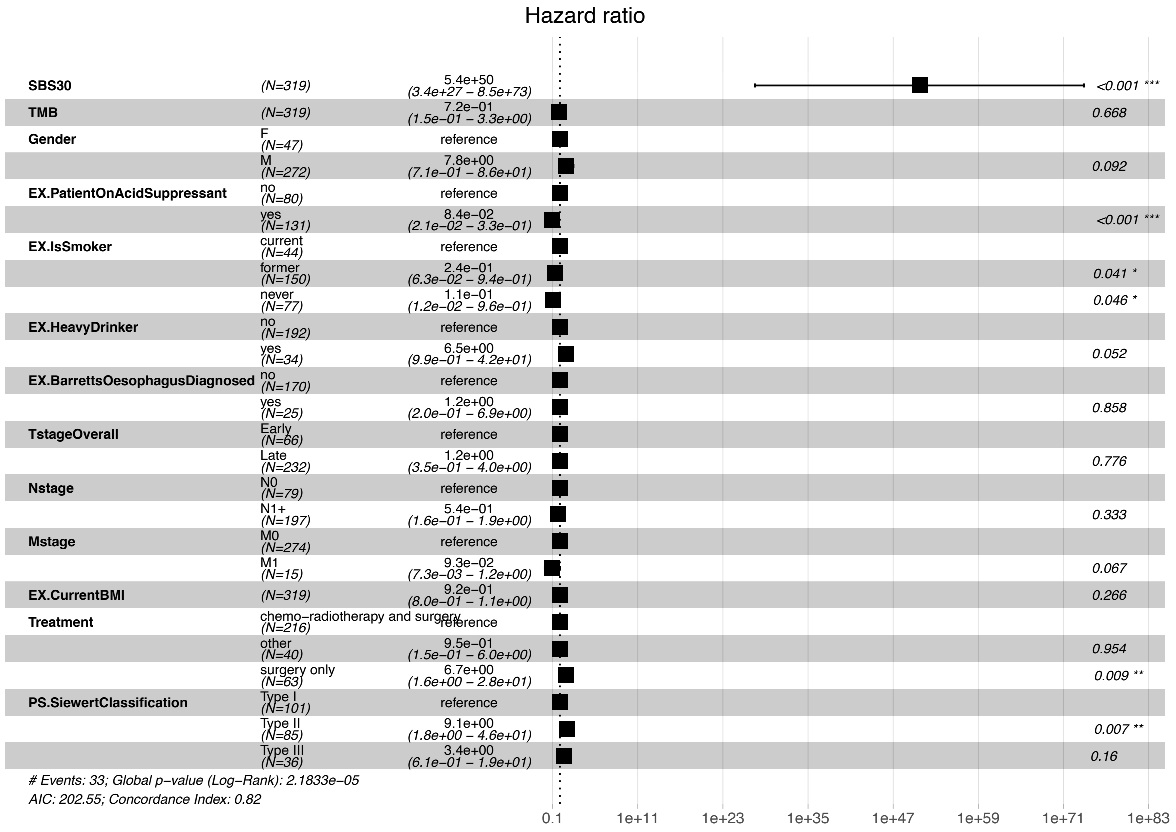

Supplementary Table 6. Overall survival outcomes modelled on SBS17b signature prevalence and clinical/demographic confounders. The results of a Cox proportional hazards model are shown (global log-rank p-value 8.8e-06). SBS17 exposure is not prognostic after accounting for cancer stage and treatment. Hazard ratio estimates are presented as boxes, with error bars indicating 95% confidence intervals.

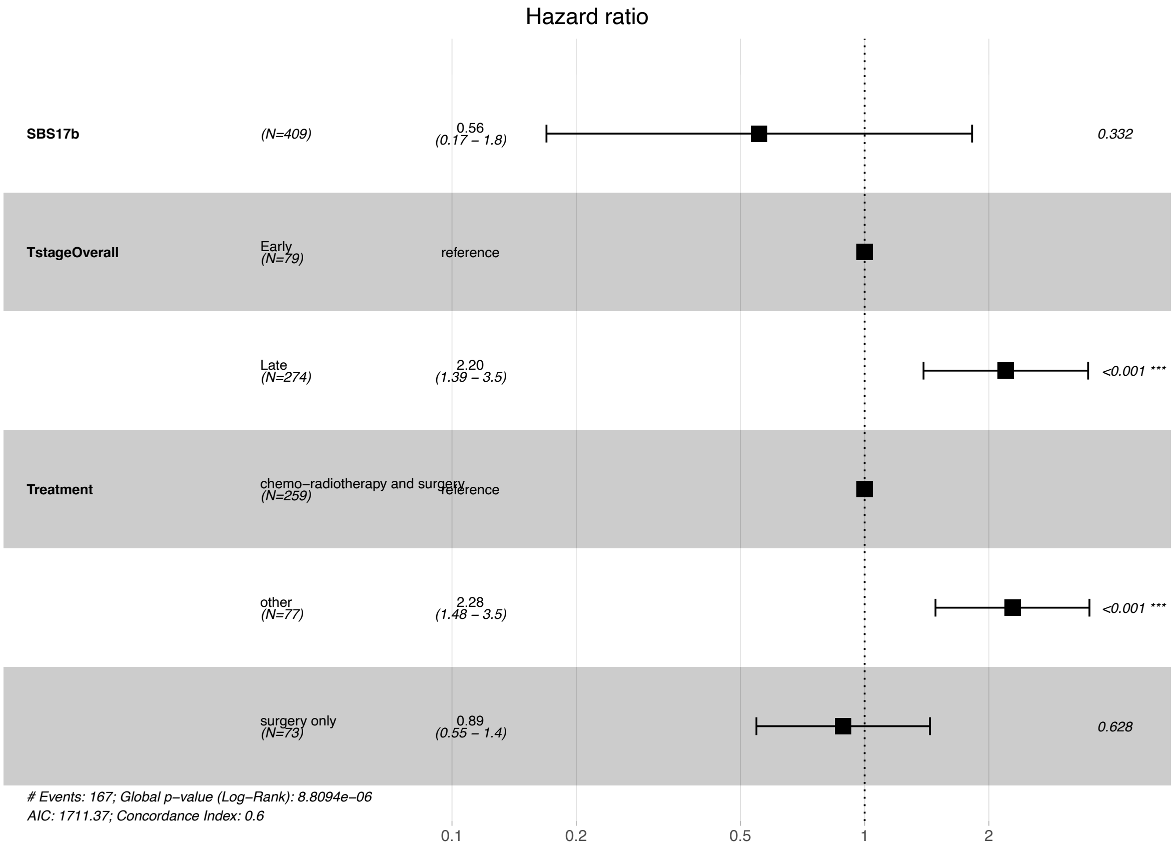

Supplementary Table 7. Summary of filters applied to single nucleotide variant (SNV) calls from Strelka. Filters were created and thresholds chosen by assessing kernel density plots of true and false positive SNV calls for a medulloblastoma International Cancer Genome Consortium (ICGC) benchmark dataset.

Filter	**Description**
DistanceToAlignmentEndMedian	The median shortest distance of the variant position within the read to either aligned end is less than 10
DistanceToAlignmentEndMAD	The median absolute deviation of the shortest distance of the variant position within the read to either aligned end is less than 3
LowMapQual	The proportion of reads at the variant position with low mapping quality (less than 1) is greater than 10%
MapQualDiffMedian	The difference in the median mapping quality of variant reads (in the tumour) and reference reads (in the normal) is greater than 5
VariantMapQualMedian	The median mapping quality of variant reads is less than 40
VariantBaseQualMedian	The median mapping quality of variant reads is less than 40
VariantAlleleCount	The number of variant-supporting reads in the tumour is less than 4
VariantAlleleCountControl	The number of variant-supporting reads in the normal is greater than 1
StrandBias	The strand bias for variant reads covering the variant position, i.e. the fraction of reads in either direction, is less than 0.02, unless the strand bias for all reads is also less than 0.02.
Repeat	The length of repetitive sequence adjacent to the variant position, where repeats can be 1-, 2-, 3-, or 4-mers, is 12 or more
SNVCluster50	The largest number of variant positions within any 50 base pair window surrounding, but excluding, the variant position is greater than 2; variant positions are those in which the number of alternate allele is supported by at least 2 reads and at least 5% of all reads covering that position.
SNVCluster100	The largest number of variant positions within any 50 base pair window surrounding, but excluding, the variant position is greater than 2; variant positions are those in which the number of alternate allele is supported by at least 2 reads and at least 5% of all reads covering that position.

SUPPLEMENTARY FIGURES

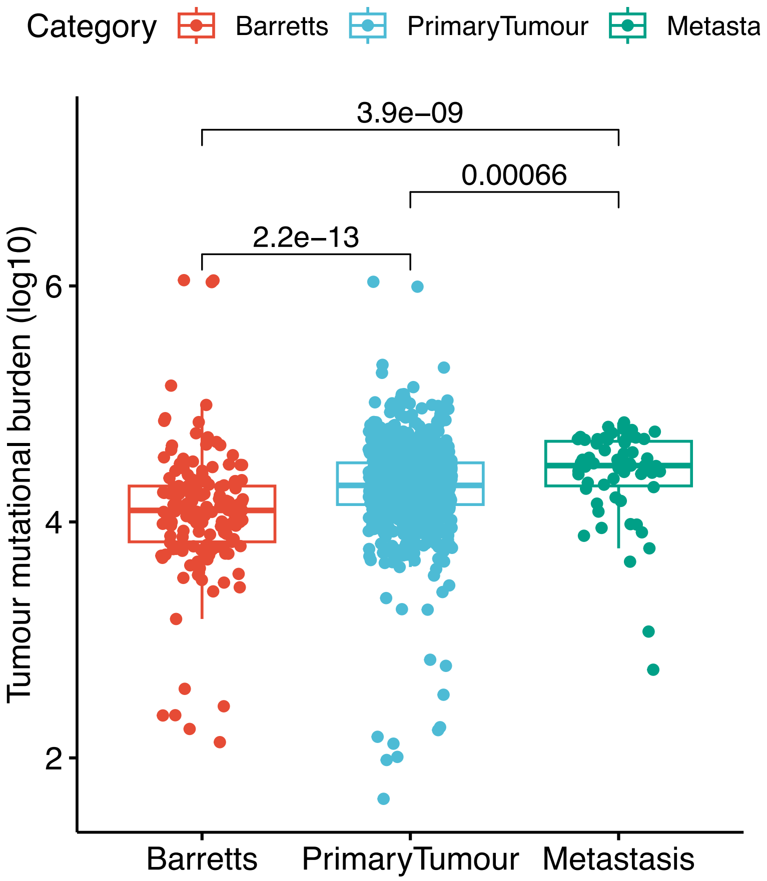

Supplementary Figure 1. Tumour mutational burden compared across disease stages (Barrett Oesophagus, n=161; primary tumours, n=777; metastases, n=59). Box boundaries represent first and third quartiles, centerline indicates median values. The upper and lower whiskers extend from the hinges to the largest and smallest values, respectively, no further than 1.5 * the inter-quartile range. Two-sided Wilcoxon signed-rank test p-values are displayed. Source data are provided as a Source Data file.

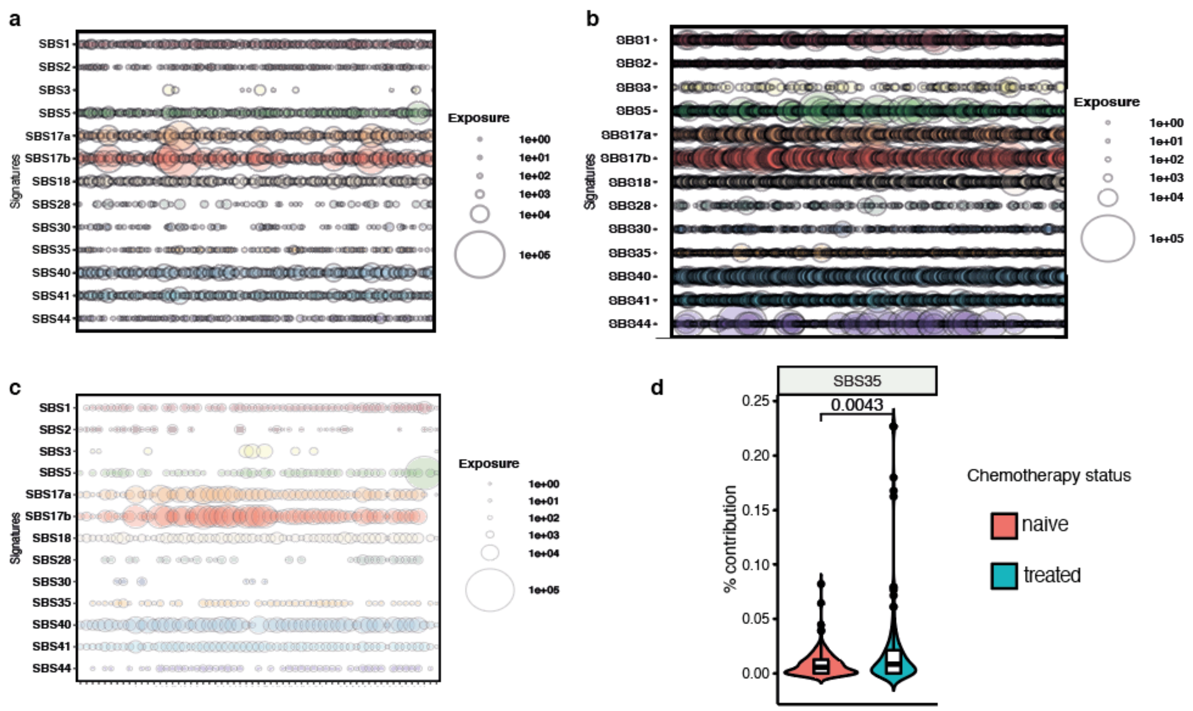

Supplementary Figure 2. Mutational signature prevalence by sample and links to chemotherapy treatment. (a-c) Landscape of mutational signature prevalence in samples at different stages of OAC development. The individual circles depict the total number of mutations attributed to various mutational signatures for each stage: (a) Barrett Oesophagus (n=161); (b) primary tumours (n=777); (c) metastases (n=59). The size of the circle is proportional to the total number of mutations linked with the respective signature in an individual sample. The x axis depicts individual samples in the cohort. (d) The contribution of the platinum-linked signature SBS35 compared between chemotherapy naïve (n=158) and treated (n=200) samples. The violin plots depict the fraction of mutations linked with this signature in chemo-naïve and treated samples. Box boundaries represent first and third quartiles, centerline indicates median values. The upper and lower whiskers extend from the hinges to the largest and smallest values, respectively, no further than 1.5 * the inter-quartile range. Outlier points are plotted individually. A two-sided Wilcoxon signed-rank test p-value is displayed. Source data are provided as a Source Data file.

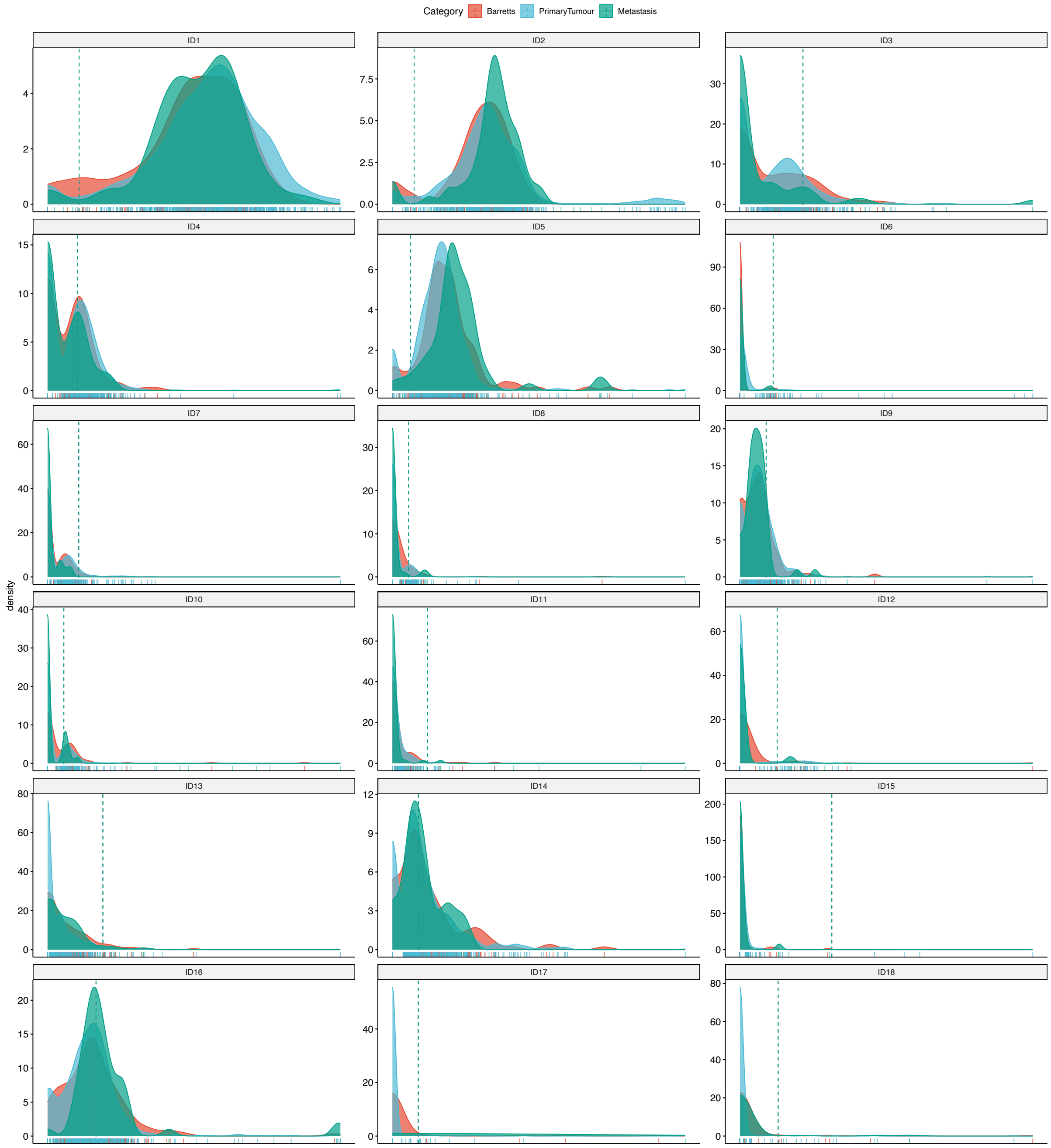

Supplementary Figure 3. Indel signature contributions in the cohort, by disease stage. The density plots depict the prevalence of indel signatures within samples of a specific disease stage. Densities are coloured according to disease stage (Barrett Oesophagus, n=161; primary tumours, n=777; metastases, n=59). Source data are provided as a Source Data file.

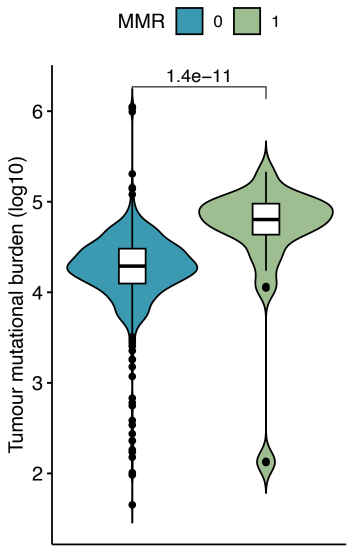

Supplementary Figure 4. Mutational burden compared between samples with (n=36) and without (n=961) MMR deficiency. Box boundaries represent first and third quartiles, centerline indicates median values. The upper and lower whiskers extend from the hinges to the largest and smallest values, respectively, no further than 1.5 * the inter-quartile range. Outlier points are plotted individually. A two-sided Wilcoxon signed-rank test p-value is displayed. Source data are provided as a Source Data file.

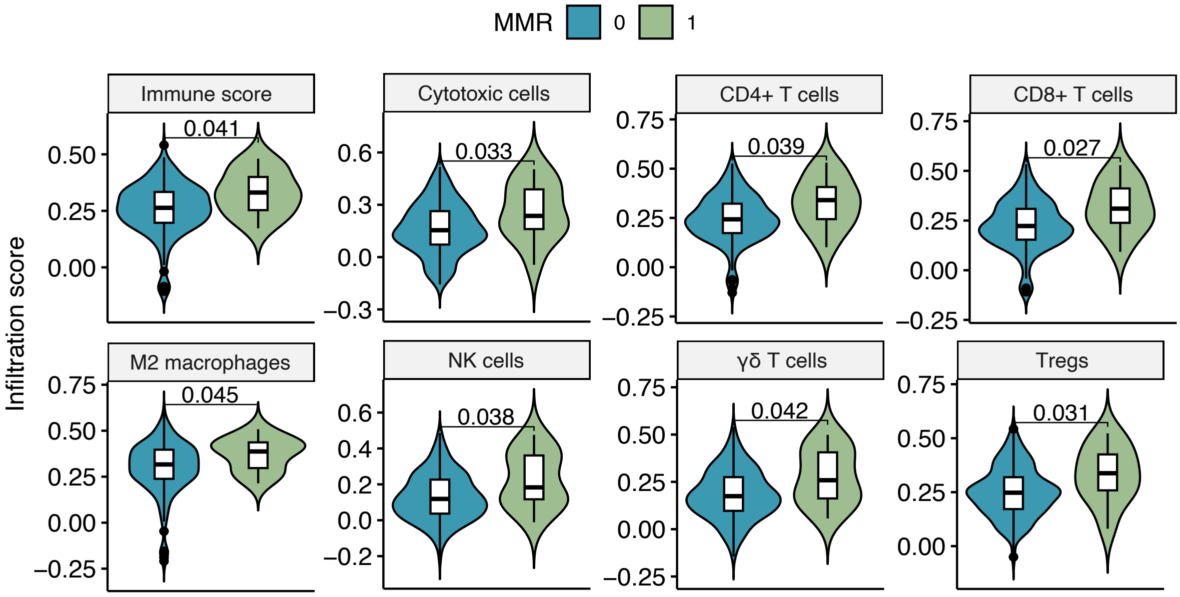

Supplementary Figure 5. Immune infiltration by MMR status. Infiltration estimates from bulk RNA-seq data using ConsensusTME (ssGSEA method) are shown for different immune cell populations and compared between samples with MMRD (n=12) and MMR proficient (n=191) samples. Box boundaries represent first and third quartiles, centerline indicates median values. The upper and lower whiskers extend from the hinges to the largest and smallest values, respectively, no further than 1.5 * the inter-quartile range. Outlier points are plotted individually. Two-sided Wilcoxon signed-rank test p-values are displayed. Source data are provided as a Source Data file.

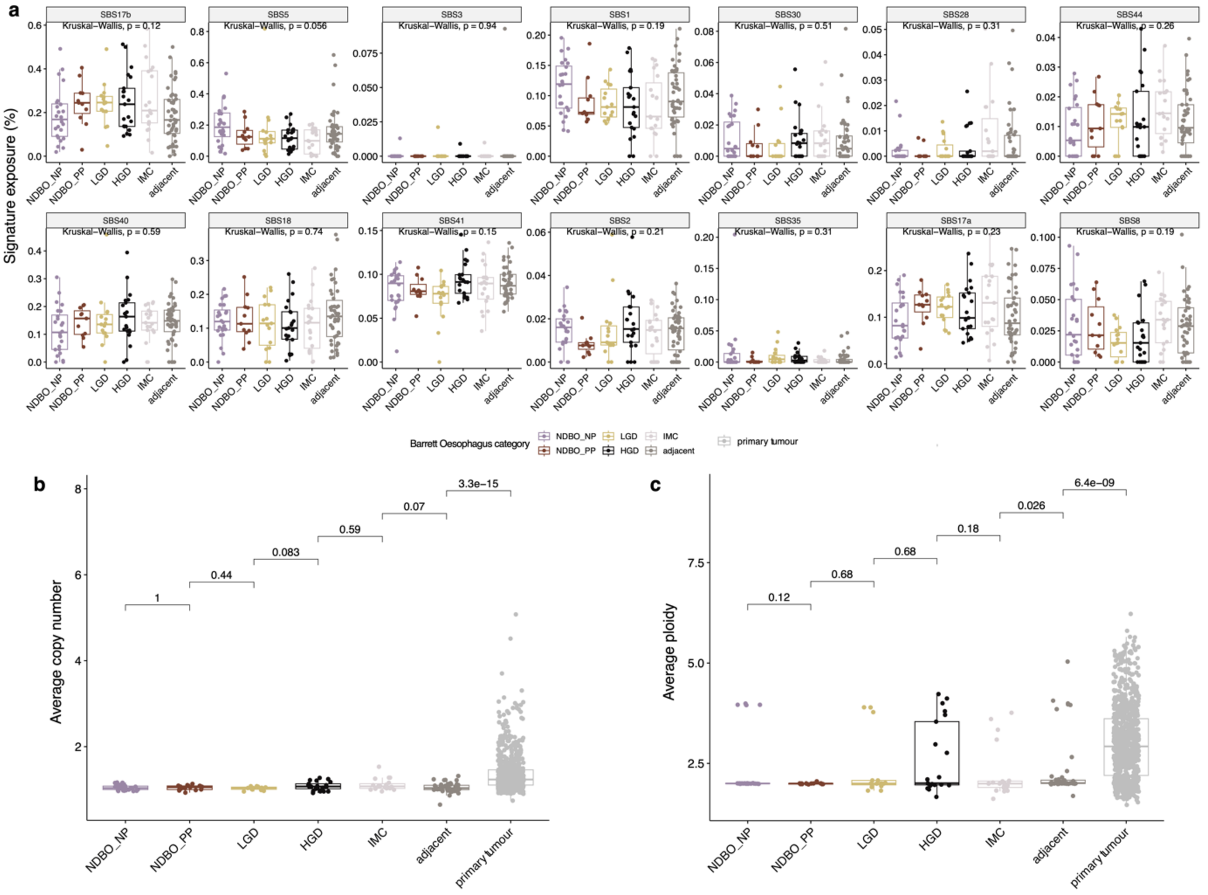

Supplementary Figure 6. Mutational signature and copy number variation by Barrett Oesophagus category. (a) Mutational signature prevalence compared between different Barrett Oesophagus samples. NDBO_NP = non-dysplastic Barrett Oesophagus, non-progressors (n=24); NDBO_PP = non-dysplastic Barrett Oesophagus, pre-progressors (n=11); LGD = low grade dysplasia (n=15); HGD = high grade dysplasia (n=21); IMC = intramucosal carcinoma (n=19); adjacent = Barrett Oesophagus adjacent to cancer (n=46). Box boundaries represent first and third quartiles, centerline indicates median values. The upper and lower whiskers extend from the hinges to the largest and smallest values, respectively, no further than 1.5 * the inter-quartile range. Kruskal-Wallis test p-values are displayed. (b) Average copy number per genome compared between different sample types. Annotations as in (a). Box boundaries represent first and third quartiles, centerline indicates median values. The upper and lower whiskers extend from the hinges to the largest and smallest values, respectively, no further than 1.5 * the inter-quartile range. Two-sided Wilcoxon signed-rank test p-values are displayed. (c) Average ploidy per genome compared between different sample types. Annotations as in (a). Box boundaries represent first and third quartiles, centerline indicates median values. The upper and lower whiskers extend from the hinges to the largest and smallest values, respectively, no further than 1.5 * the inter-quartile range. Outlier points are plotted individually. Two-sided Wilcoxon signed-rank test p-values are displayed. Source data are provided as a Source Data file.

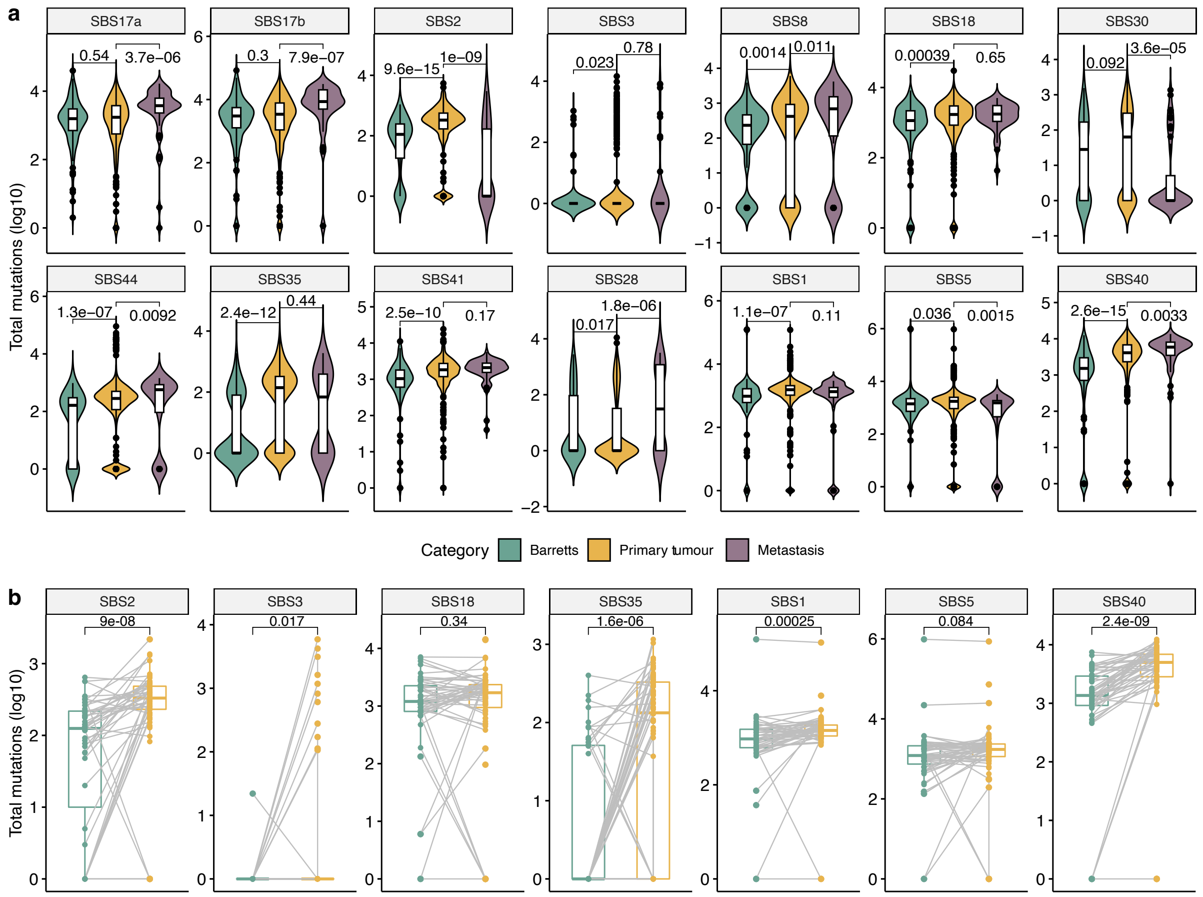

Supplementary Figure 7. Variations in total mutation contribution by various signatures from Barrett Oesophagus to primary tumours and metastases. Absolute numbers of mutations are compared between states. (a) Mutational signature contributions compared across the three disease conditions in non-matched samples (114 Barrett Oesophagus, 706 primary tumours, 55 metastases). Box boundaries represent first and third quartiles, centerline indicates median values. The upper and lower whiskers extend from the hinges to the largest and smallest values, respectively, no further than 1.5 * the inter-quartile range. Outlier points are plotted individually. Two-sided Wilcoxon signed-rank test p-values are displayed. (b) Changes in mutational signature prevalence between matched Barrett Oesophagus and primary tumour samples (n=47). Blue triangles denote an increase in signature contribution in primary tumours; red triangles denote a decrease. Only signatures with a significant change are shown. Box boundaries represent first and third quartiles, centerline indicates median values. The upper and lower whiskers extend from the hinges to the largest and smallest values, respectively, no further than 1.5 * the inter-quartile range. Outlier points are plotted individually. Two-sided Wilcoxon signed-rank test p-values are displayed. Source data are provided as a Source Data file.

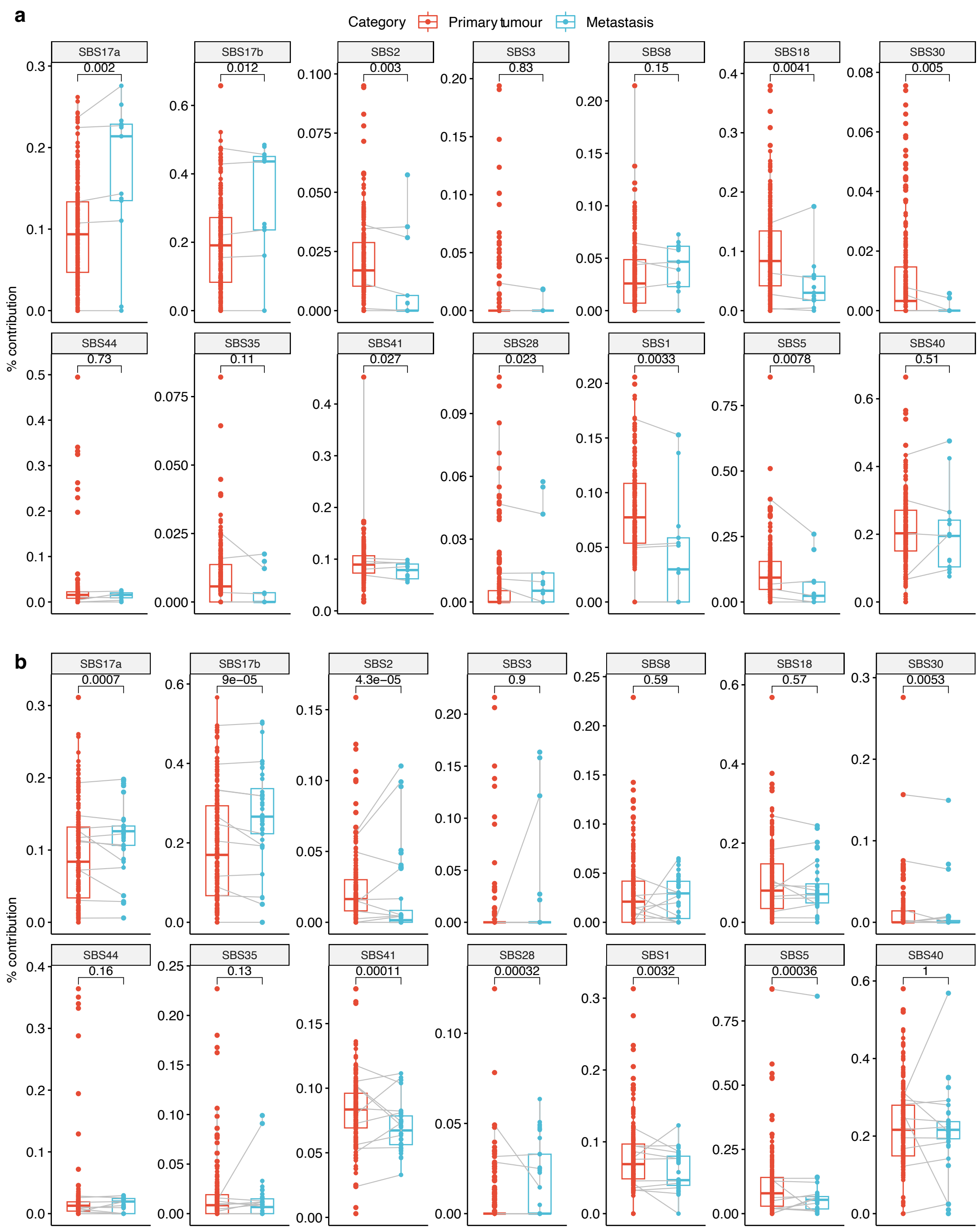

Supplementary Figure 8. Relative mutational contributions from various signatures to primary tumours and metastases in (a) treatment-naïve (221 primary tumours, 13 metastases); (b) chemotherapy treated samples (189 primary tumours, 44 metastases). The relative number of mutations contributed by each signature is compared between the two disease stages. Matched cases are linked with a grey line. Box boundaries represent first and third quartiles, centerline indicates median values. The upper and lower whiskers extend from the hinges to the largest and smallest values, respectively, no further than 1.5 * the inter-quartile range. Two-sided Wilcoxon signed-rank test p-values are displayed. Source data are provided as a Source Data file.

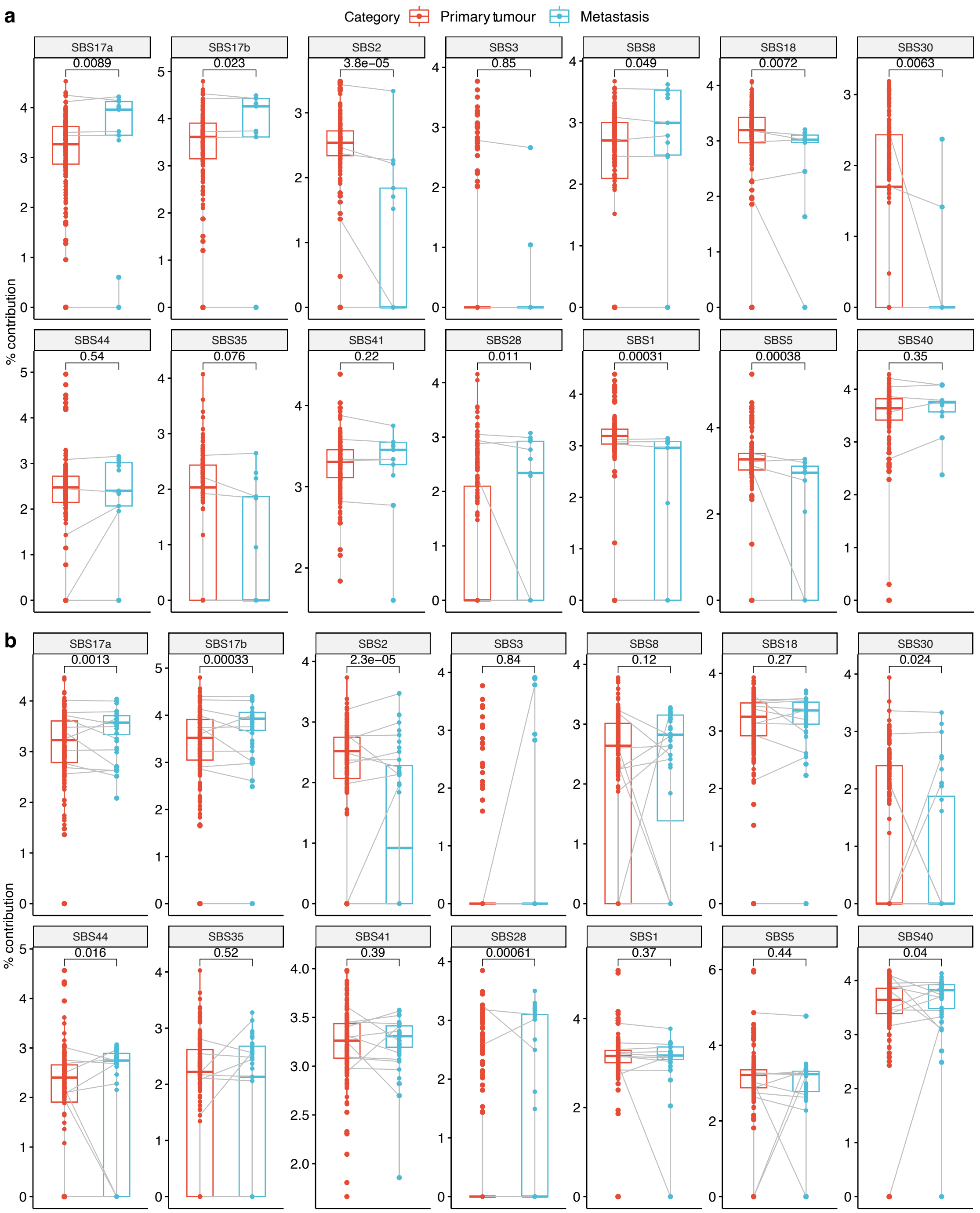

Supplementary Figure 9. Absolute mutational contributions from various signatures to treatment naïve primary tumours and metastases in (a) treatment-naïve (221 primary tumours, 13 metastases); (b) chemotherapy treated samples (189 primary tumours, 44 metastases). The absolute number of mutations (log10) contributed by each signature is compared between the two disease stages. Matched cases are linked with a grey line. Box boundaries represent first and third quartiles, centerline indicates median values. The upper and lower whiskers extend from the hinges to the largest and smallest values, respectively, no further than 1.5 * the inter-quartile range. Two-sided Wilcoxon signed-rank test p-values are displayed. Source data are provided as a Source Data file.

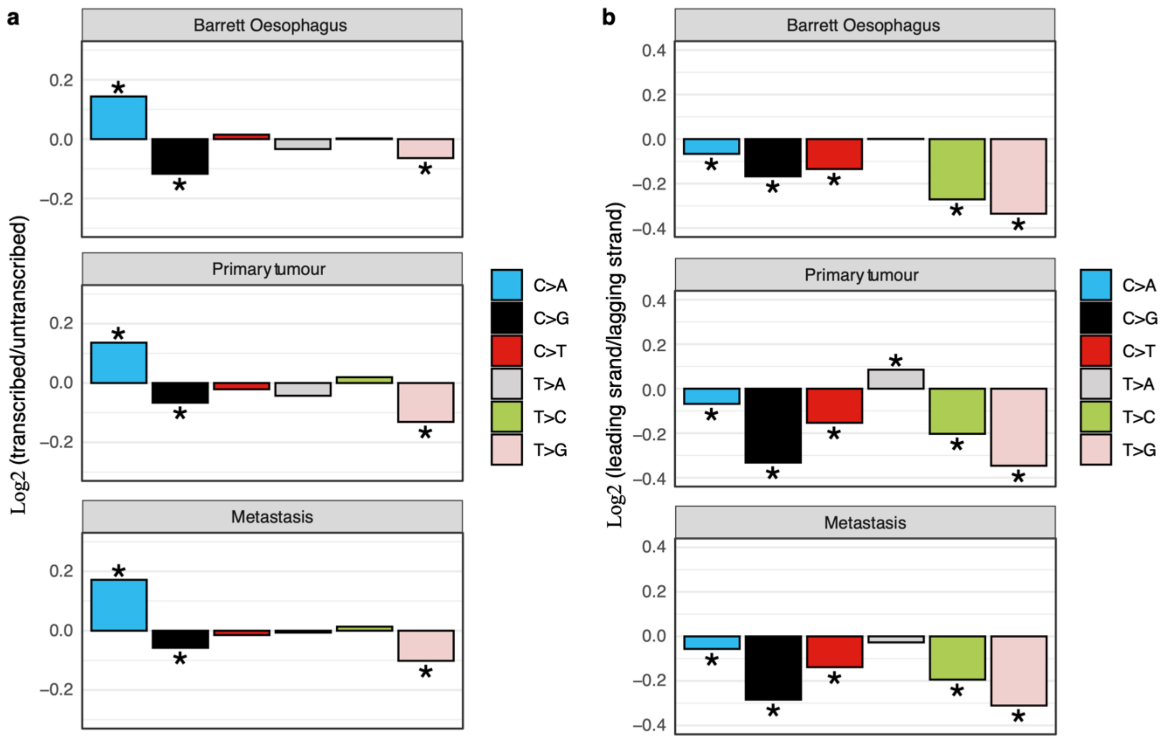

Supplementary Figure 10. Transcription and replication strand asymmetry in mutational accumulation across different stages of OAC development. SNVs (C > X / T > X) were mapped to the transcribed/untranscribed strands and leading /lagging replicative strands. Significant strand asymmetries are marked by asterisks (one-sided Poisson test FDR-adjusted for multiple comparisons across 25 randomly sampled genomes in every category). (a) T>G mutations (signature 17 associated) were enriched on the untranscribed strand across Barretts, OAC and metastases. (b) The lagging replicative strand is mapped by all types of substitutions, with T>G and T>C substitutions predominantly present on lagging strand across Barretts, OAC and metastases. Source data are provided as a Source Data file, including exact p-values for every category.

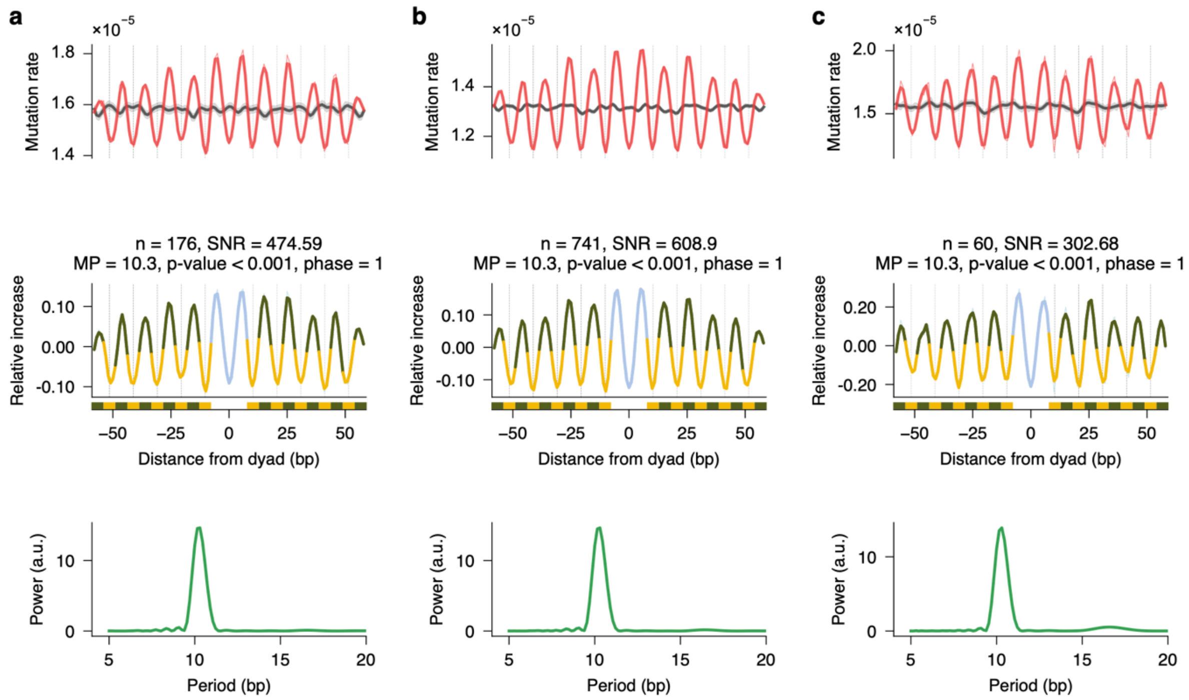

Supplementary Figure 11. Mutation rate periodicity across nucleosome-covered DNA stretches, compared between minor groove facing histones (minor-in) and minor groove facing away from them (minor-out). The observed and expected mutation rate of sequenced samples (top), relative increase of mutation rate (middle), and its periodogram (bottom) are shown for: (a) Barrett Oesophagus cases, (b) primary tumours, and (c) metastases. Vertical broken lines represent stretches of minor groove facing away from histones. Yellow in middle plot indicates minor groove facing away from histones (minor-in) and green indicates minor groove facing histones (minor-out). The empirical p value of the relative increase of mutation rate is inferred by counting the number of permutations with expected SNR above the observed SNR (one-sided Student's t test, adjusted for multiple testing using the Benjamini-Hochberg method, p=0.001). The error bar corresponds to +/- the standard deviation of the randomisations per position. Source data are provided as a Source Data file.

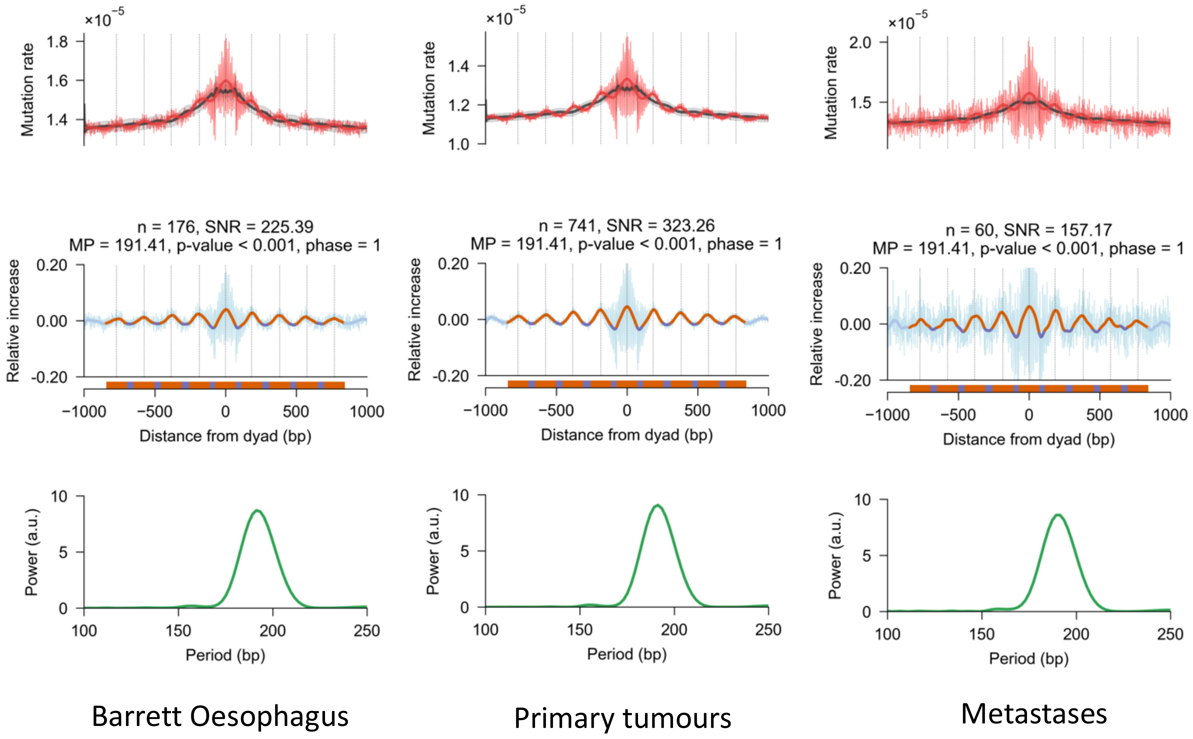

Supplementary Figure 12. Mutation rate periodicity between nucleosome-covered and linker DNA. The first set of panels depicts all Barrett Oesophagus cases, the second primary tumours, and the third refers to metastases. (p-value = 0.001, considering an empirical p-value calculated out of 1000 randomisations). The empirical p value of the relative increase of mutation rate is inferred by counting the number of permutations with expected SNR above the observed SNR (one-sided Student's t test, adjusted for multiple testing using the Benjamini-Hochberg method, p=0.001). The error bar corresponds to +/- the standard deviation of the randomisations per position. Source data are provided as a Source Data file.

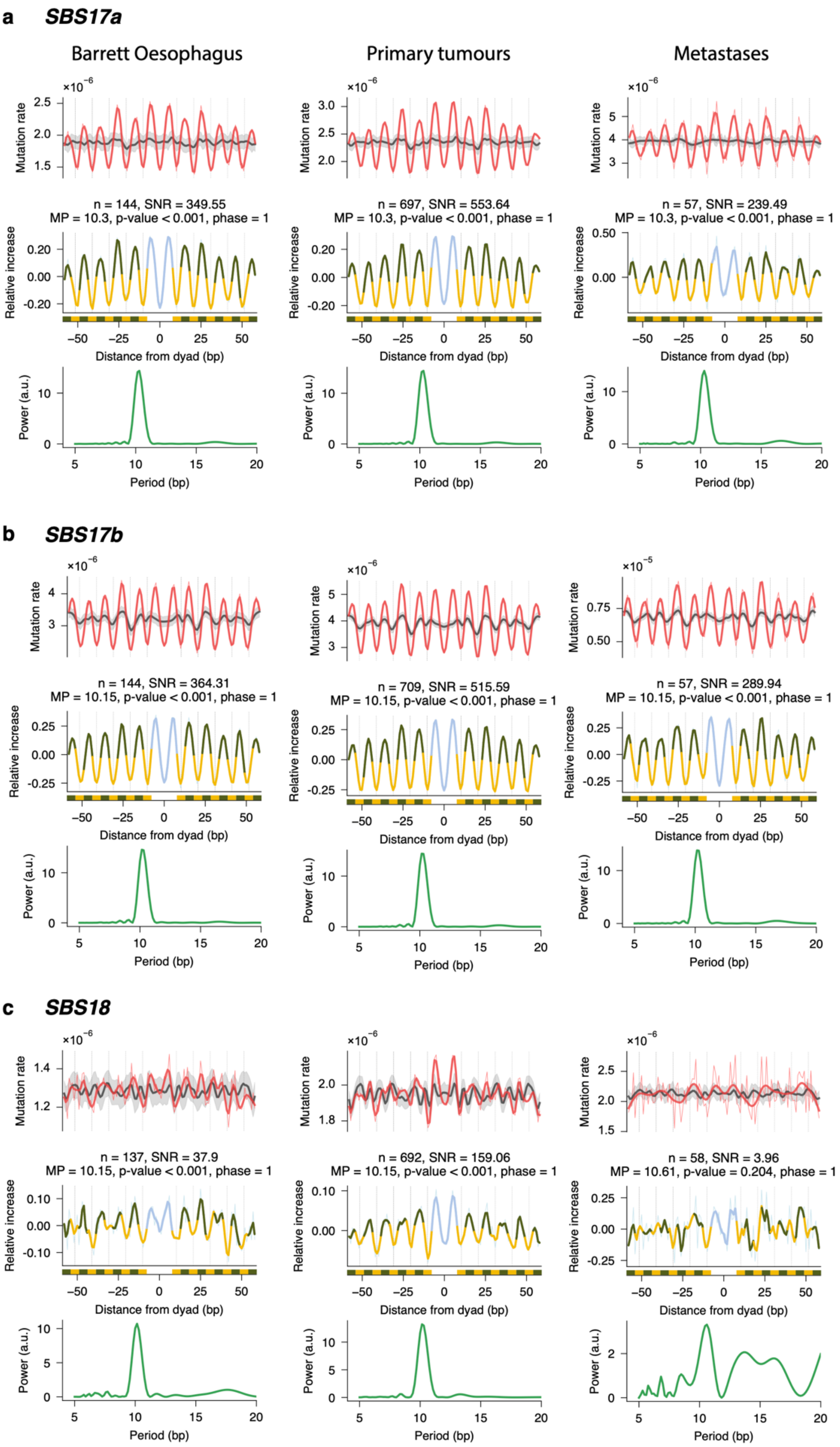

Supplementary Figure 13. Mutation rate periodicity for selected mutational signatures across nucleosome-covered DNA stretches, compared between minor groove facing histones (minor-in) and minor groove facing away from them (minor-out). The periodicity

pattern is shown for mutations associated with (a) SBS17a, (b) SBS17b, and (c) SBS18, for Barrett Oesophagus (left), primary tumours (center) and metastases (right). The observed and expected mutation rate of sequenced samples (top), relative increase of mutation rate (middle), and its periodogram (bottom) are shown. Vertical broken lines represent stretches of minor groove facing away from histones. Yellow in middle plot indicates minor groove facing away from histones (minor-in) and green indicates minor groove facing histones (minor-out). The empirical p value of the relative increase of mutation rate is inferred by counting the number of permutations with expected SNR above the observed SNR (one-sided Student's t test, adjusted for multiple testing using the Benjamini-Hochberg method, p=0.001). The error bar corresponds to +/- the standard deviation of the randomisations per position.
Source data are provided as a Source Data file.

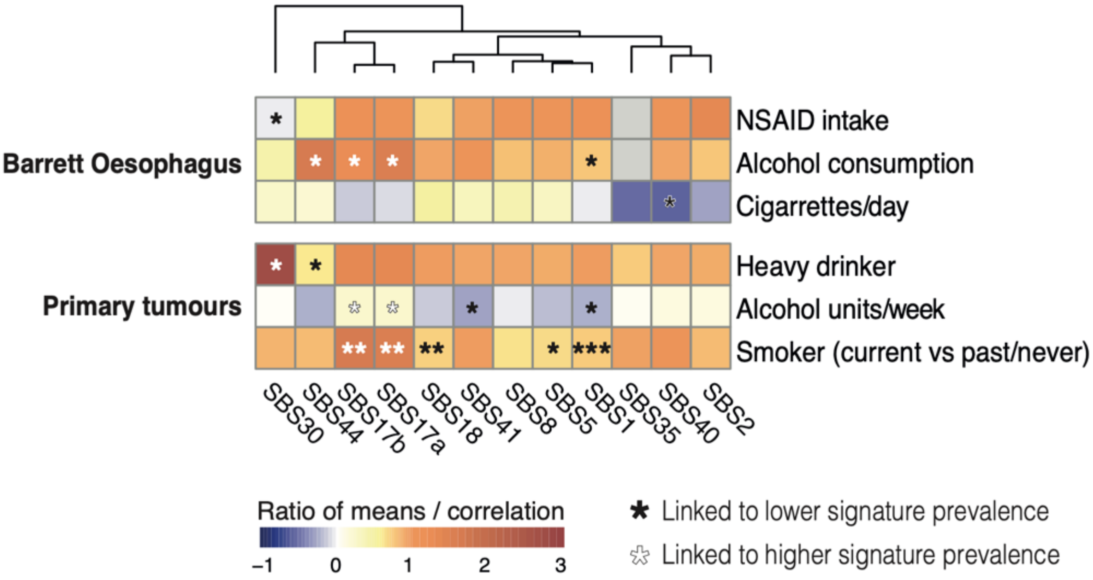

Supplementary Figure 14. Correlation between mutational signature prevalence in Barrett Oesophagus and primary tumours and risk factors of oesophageal adenocarcinoma. The heat map colour gradient indicates the ratio of median exposures between the groups with/without the risk factor (for binary factors, two-sided Wilcoxon rank-sum test) or the two-sided Pearson correlation coefficient (for continuous factors, i.e. Barrett length, cigarettes/day and alcohol units/week). Asterisks are displayed where a significant association was found: *p<0.05, **p<0.01; ***p<0.001. White asterisks indicate an increase in signature exposure is associated with the risk factor; black asterisks correspond to a decrease in exposure. Exact p-values from left to right, top to bottom: 0.031, 0.022, 0.04, 0.022,0.025, 0.042 (Barrett Oesophagus), 0.046, 0.0024, 0.026, 0.028, 0.0072, 0.043, 0.0035, 0.0026, 0.0092, 0.014, 0.00042 (primary tumours). Source data are provided as a Source Data file.

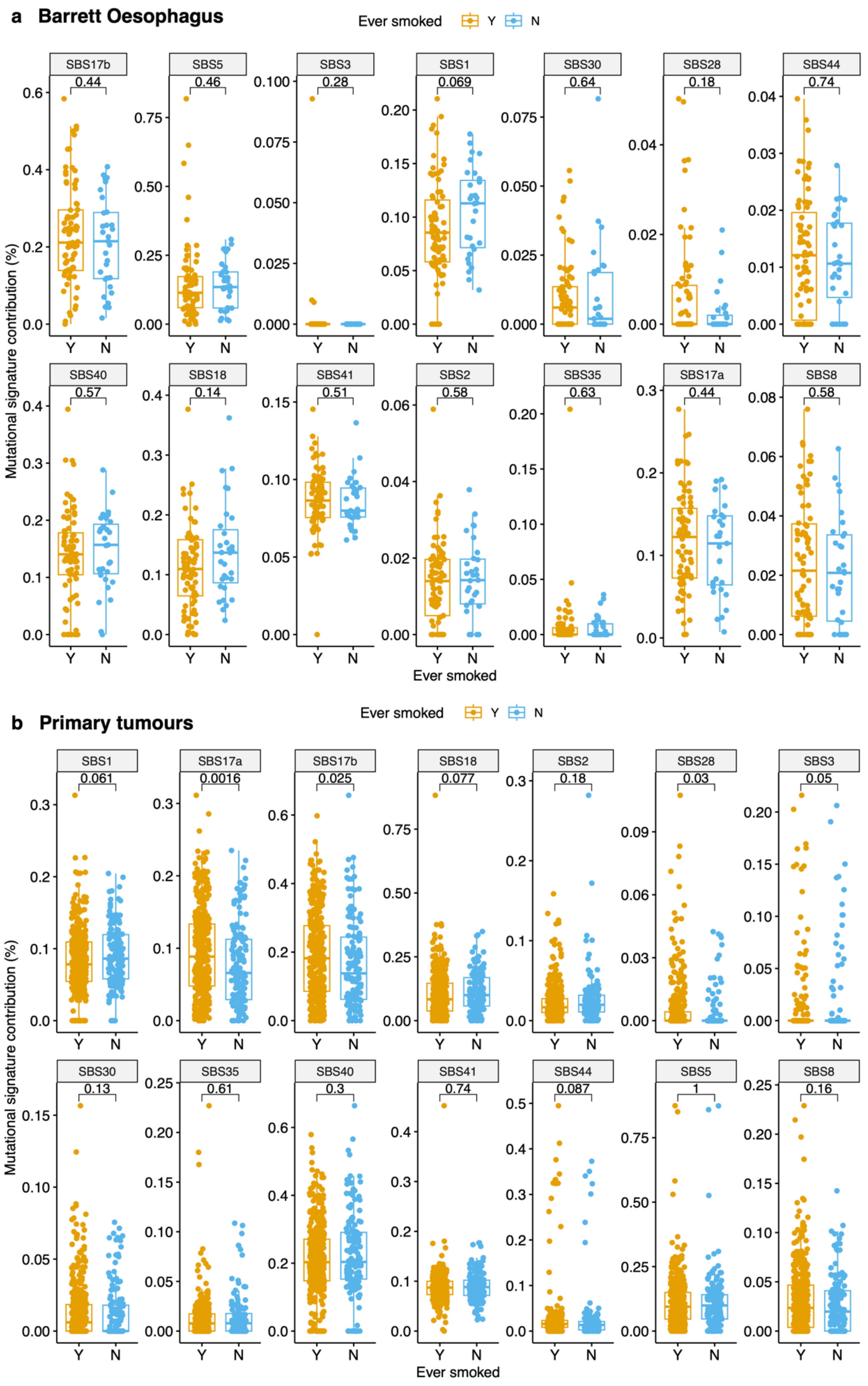

Supplementary Figure 15. Mutational signature exposures compared between smokers and never smokers. Mutational signature contributions are compared between past/present

smokers (Y) and never smokers (N) within (a) Barrett Oesophagus (79 smokers, 31 never smokers) and (b) primary tumour samples (365 smokers, 143 never smokers). Box boundaries represent first and third quartiles, centerline indicates median values. The upper and lower whiskers extend from the hinges to the largest and smallest values, respectively, no further than 1.5 * the inter-quartile range. Two-sided Wilcoxon signed-rank test p-values are displayed. Source data are provided as a Source Data file.

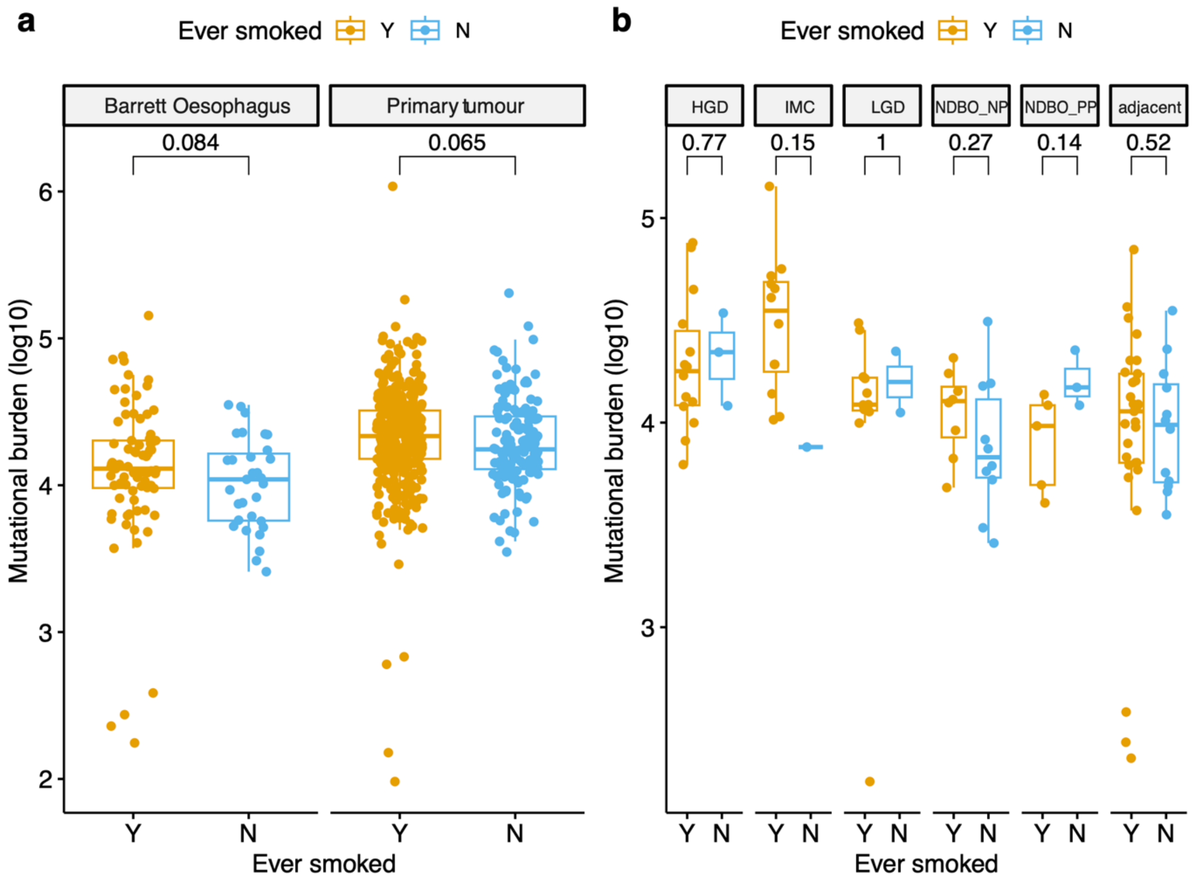

Supplementary Figure 16. Mutational burden compared between smokers and never smokers. (a) Mutational loads per genome are compared between past/present (Y) and never (N) smokers within Barrett Oesophagus (79 Y, 31 N) and primary tumour (365 Y, 143 N) cases. Box boundaries represent first and third quartiles, centerline indicates median values. The upper and lower whiskers extend from the hinges to the largest and smallest values, respectively, no further than 1.5 * the inter-quartile range. Two-sided Wilcoxon signed-rank test p-values are displayed. (b) Mutational loads are compared between past/present (Y) and never (N) smokers within different Barrett Oesophagus categories: NDBO_NP = non-dysplastic Barrett Oesophagus, non-progressors; NDBO_PP = non-dysplastic Barrett Oesophagus, pre-progressors (8 Y, 10 N (11 Y, 2N); HGD = high grade dysplasia (14 Y, 3N); IMC = intramucosal carcinoma (12 Y, 1N); adjacent = Barrett Oesophagus adjacent to cancer (29Y, 12N). Box boundaries represent first and third quartiles, centerline indicates median values. The upper and lower whiskers extend from the hinges to the largest and smallest values, respectively, no further than 1.5 * the inter-quartile range. Two-sided Wilcoxon signed-rank test p-values are displayed. Source data are provided as a Source Data file.

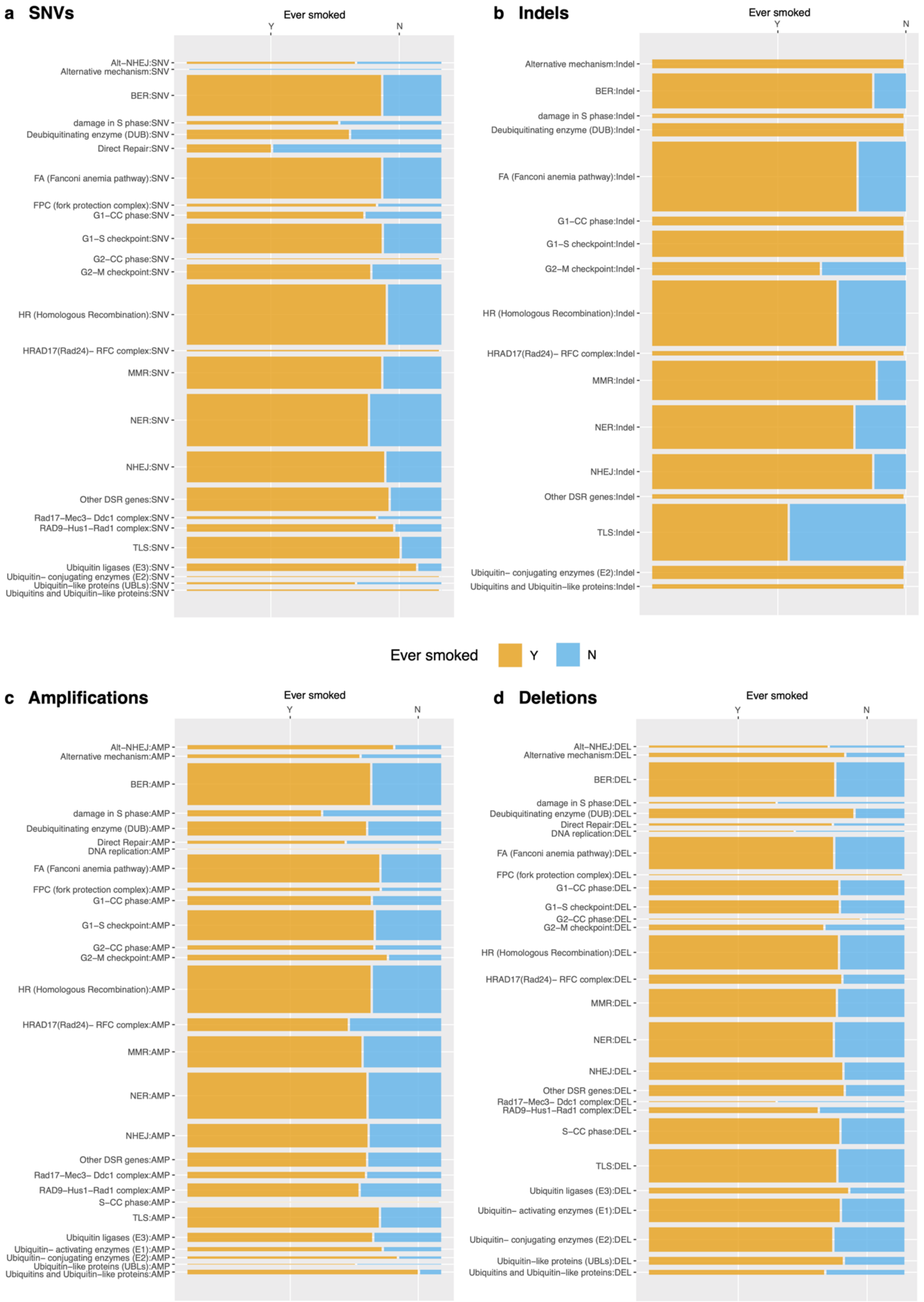

Supplementary Figure 17. Genomic changes in DNA damage repair pathways in smokers versus non-smokers. The mosaic plots display the fractions of past/present (Y) and never (N) smokers harbouring (a) SNVs, (b) indels, (c) copy number amplifications, and (d) deletions in various DNA damage repair pathways as listed on the y axis. Source data are provided as a Source Data file.

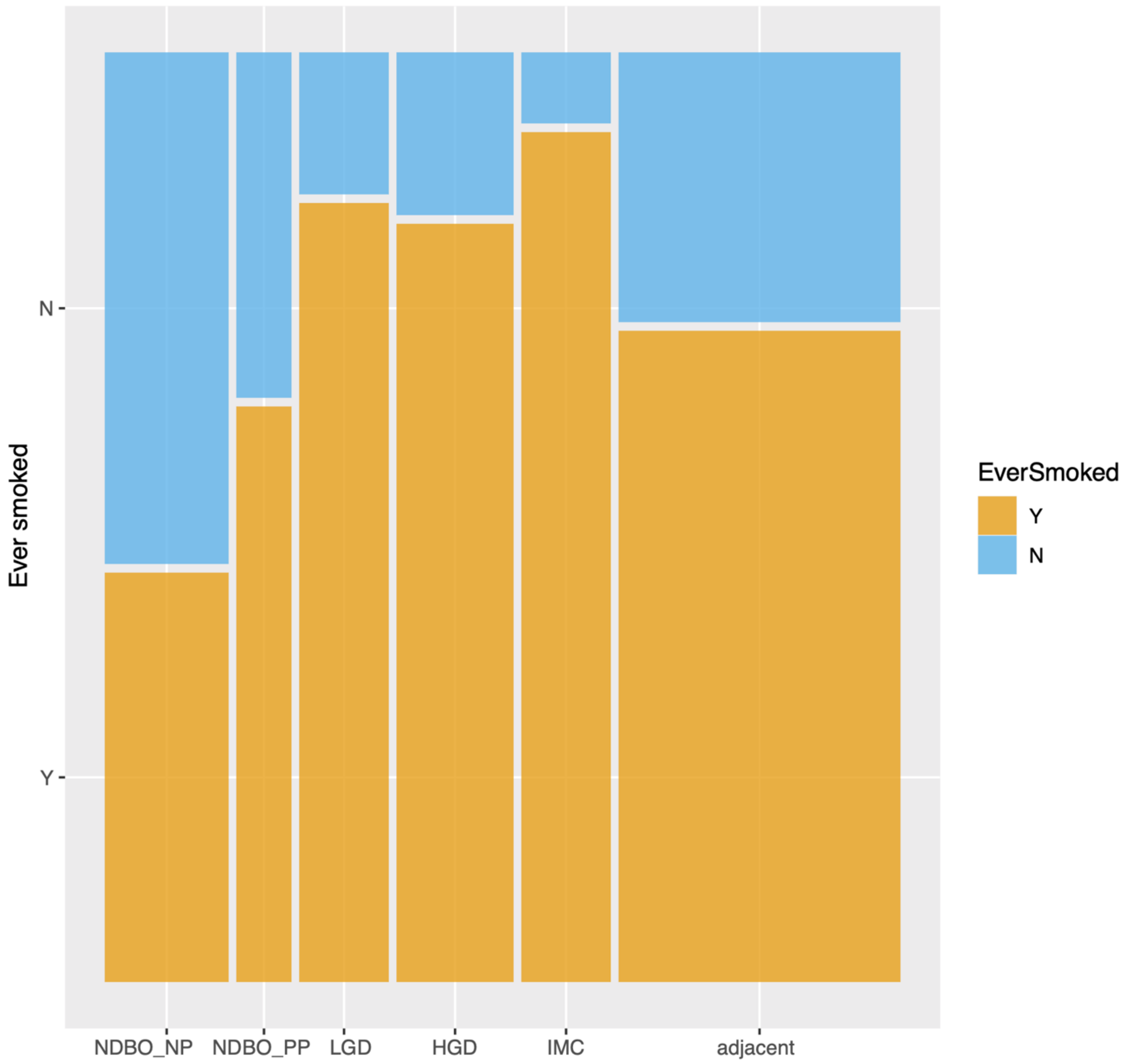

Supplementary Figure 18. Breakdown of smokers and never smokers in the Barrett Oesophagus cohort. The mosaic plot shows the fraction of samples taken from past/present (Y) and never (N) smokers belonging to different Barrett Oesophagus categories: NDBO_NP = non-dysplastic Barrett Oesophagus, non-progressors; NDBO_PP = non-dysplastic Barrett Oesophagus, pre-progressors; LGD = low grade dysplasia; HGD = high grade dysplasia; IMC = intramucosal carcinoma; adjacent = Barrett Oesophagus adjacent to cancer. Source data are provided as a Source Data file.

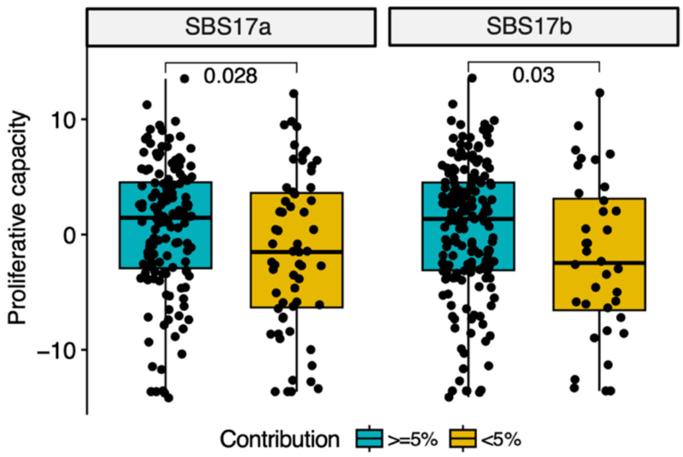

Supplementary Figure 19. Proliferative capacity of tumours with/without SBS17 mutagenesis. Tumours with evidence for SBS17a and SBS17b mutagenesis present increased proliferative capacity. The box plots compare biologically independent samples with SBS17a contribution >5% (n=144) versus <5%(n=59), and SBS17b contribution >5% (n=167) versus <5% (n=36). Box boundaries represent first and third quartiles, centerline indicates median values. The upper/lower whiskers extend from the hinges to the largest and smallest values, respectively, no further than 1.5 * the inter-quartile range. Two-sided Wilcoxon signed-rank test p-values are displayed. Source data are provided as a Source Data file.

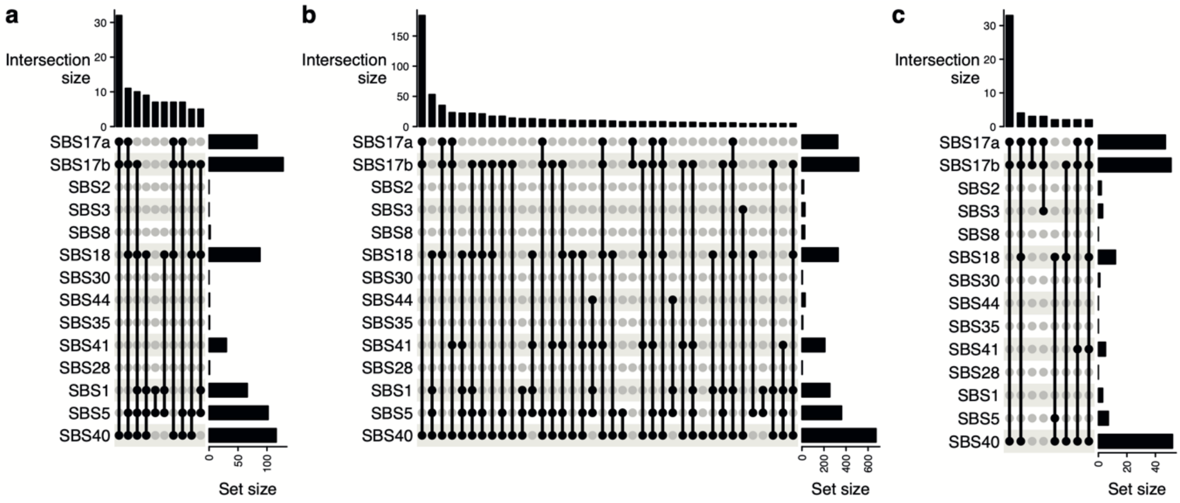

Supplementary Figure 20. Signature combinations found across the spectrum of oesophageal cancer development. (a-c) The upset plots indicate mutational signatures that are jointly present in sequenced samples from (a) Barrett Oesophagus, (b) primary oesophageal cancer and (c) oesophageal metastases, along with their individual (set size) and joint frequencies (intersection size). Only combinations of signatures contributing at least 10% of the mutations in a sample are shown. Source data are provided as a Source Data file.

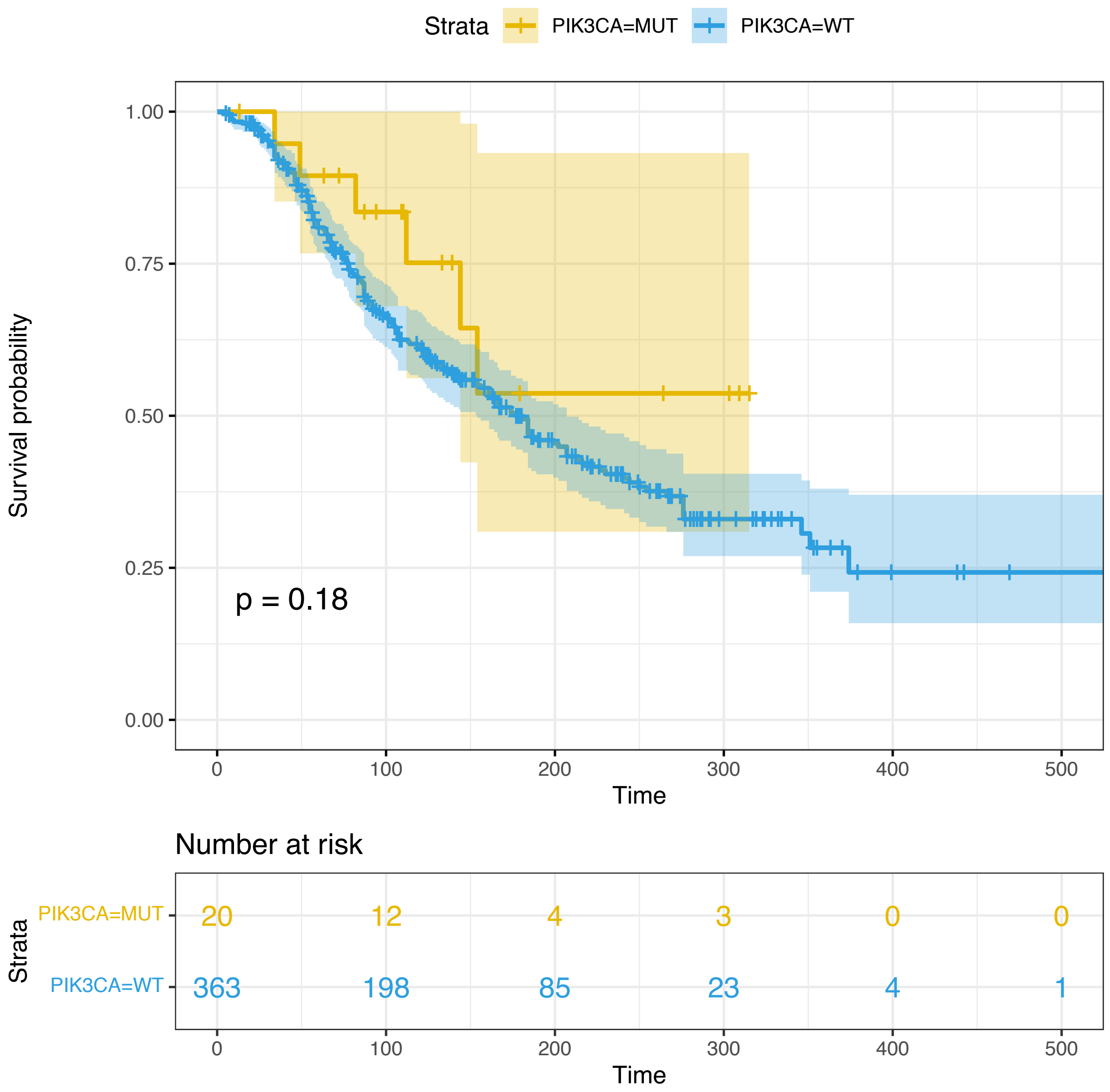

Supplementary Figure 21. Overall survival of patients with and without PIK3CA mutations. The results of a Cox proportional hazards analysis are displayed, with the log-rank test p-value reported. The shaded areas depict the 95% confidence intervals. The number of patients at risk at various time intervals are shown in the table below. Source data are provided as a Source Data file.

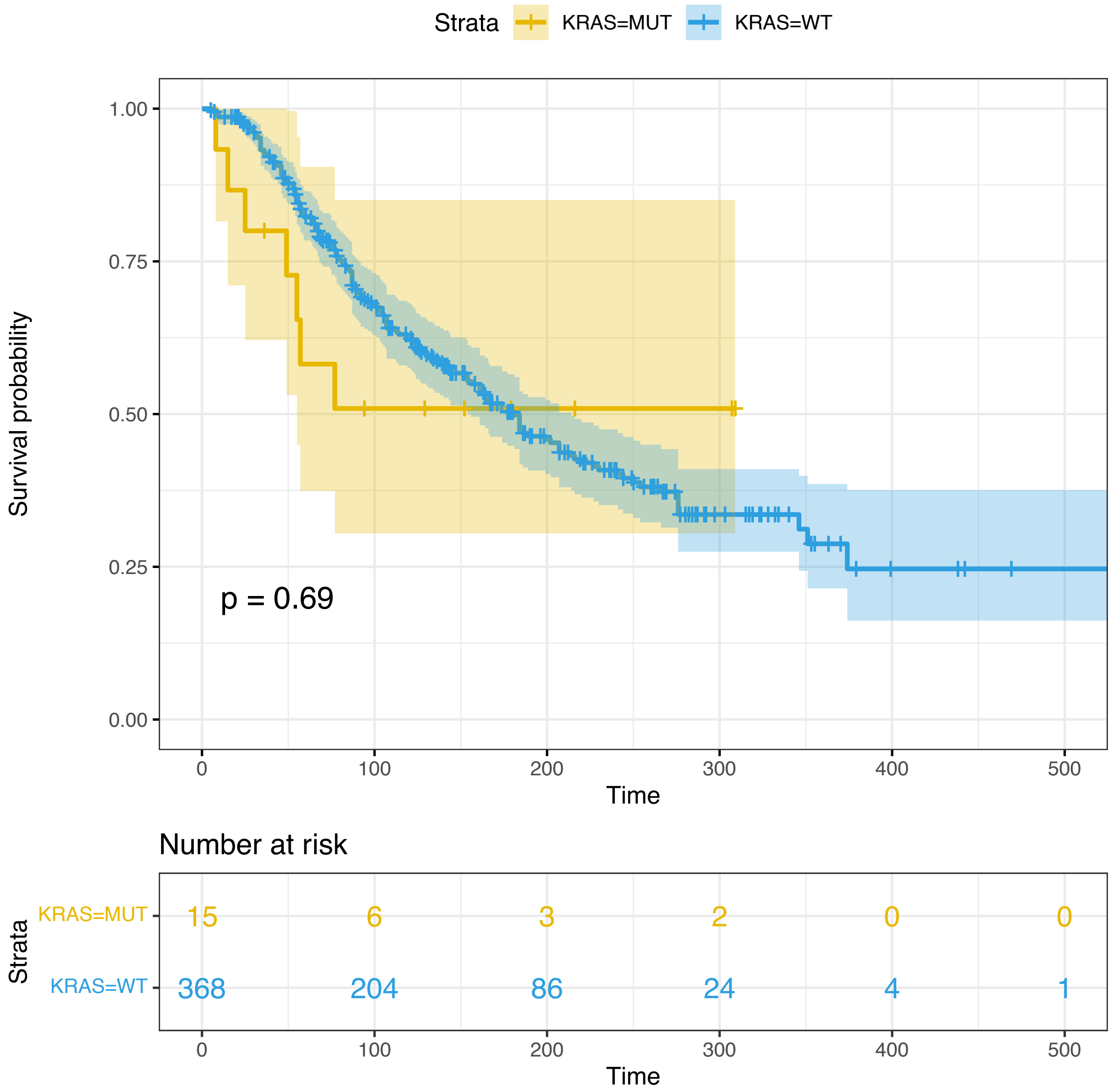

Supplementary Figure 22. Overall survival of patients with and without KRAS mutations. The results of a Cox proportional hazards analysis are displayed, with the log-rank test p-value reported. The shaded areas depict the 95% confidence intervals. The number of patients at risk at various time intervals are shown in the table below. Source data are provided as a Source Data file.

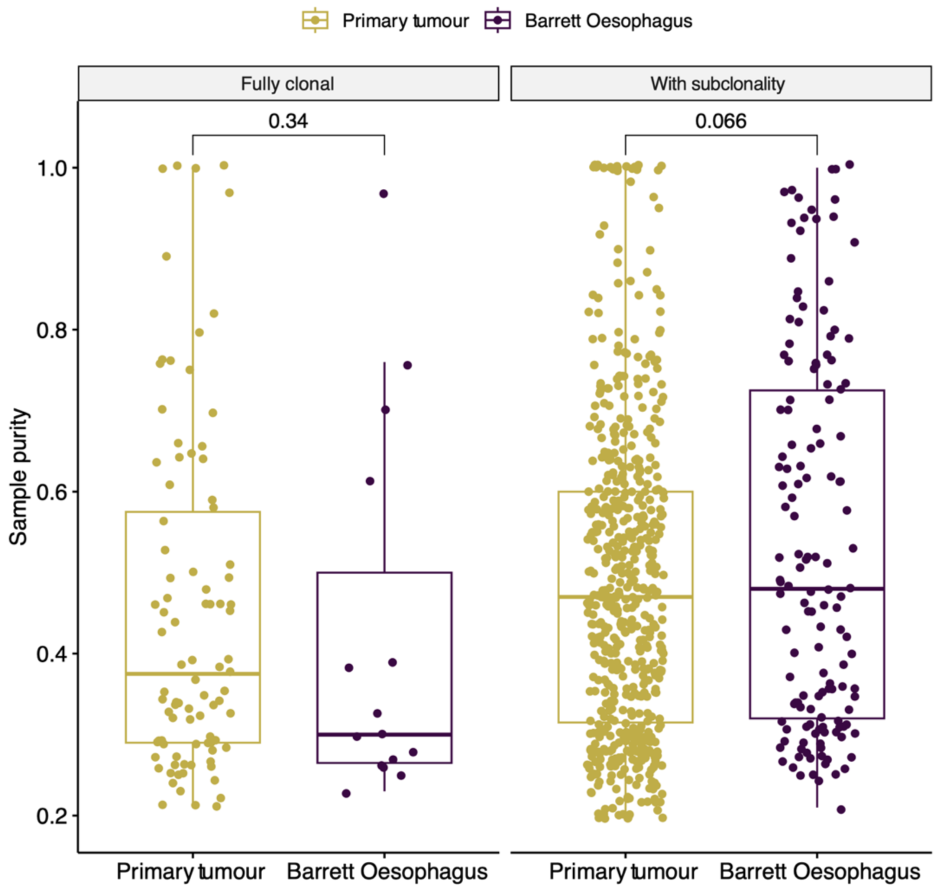

Supplementary Figure 23. Tumour purity variation within Barrett Oesophagus and primary tumour samples, by subclonality.

Left: Primary tumour (n=90) and Barrett Oesophagus (n=15) sample purities are compared within fully clonal samples. Box boundaries represent first and third quartiles, centerline indicates median values. The upper and lower whiskers extend from the hinges to the largest and smallest values, respectively, no further than 1.5 * the inter-quartile range. Two-sided Wilcoxon signed-rank test p-values comparing fully clonal primary tumours and Barrett samples are displayed.

Right: Primary tumour (n=579) and Barrett Oesophagus (n=142) sample purities are compared within samples showing evidence for subclonality. Box boundaries represent first and third quartiles, centerline indicates median values. The upper and lower whiskers extend from the hinges to the largest and smallest values, respectively, no further than 1.5 * the inter-quartile range. Two-sided Wilcoxon signed-rank test p-values comparing primary tumours and Barrett samples with evidence of subclonality are displayed. While there is a slight decrease in purity for fully clonal samples, potentially due to higher immune recognition/infiltration of a more homogeneous cancer entity, the range of purity is similar between primary tumours and BO samples regardless of subclonality.

Source data are provided as a Source Data file.

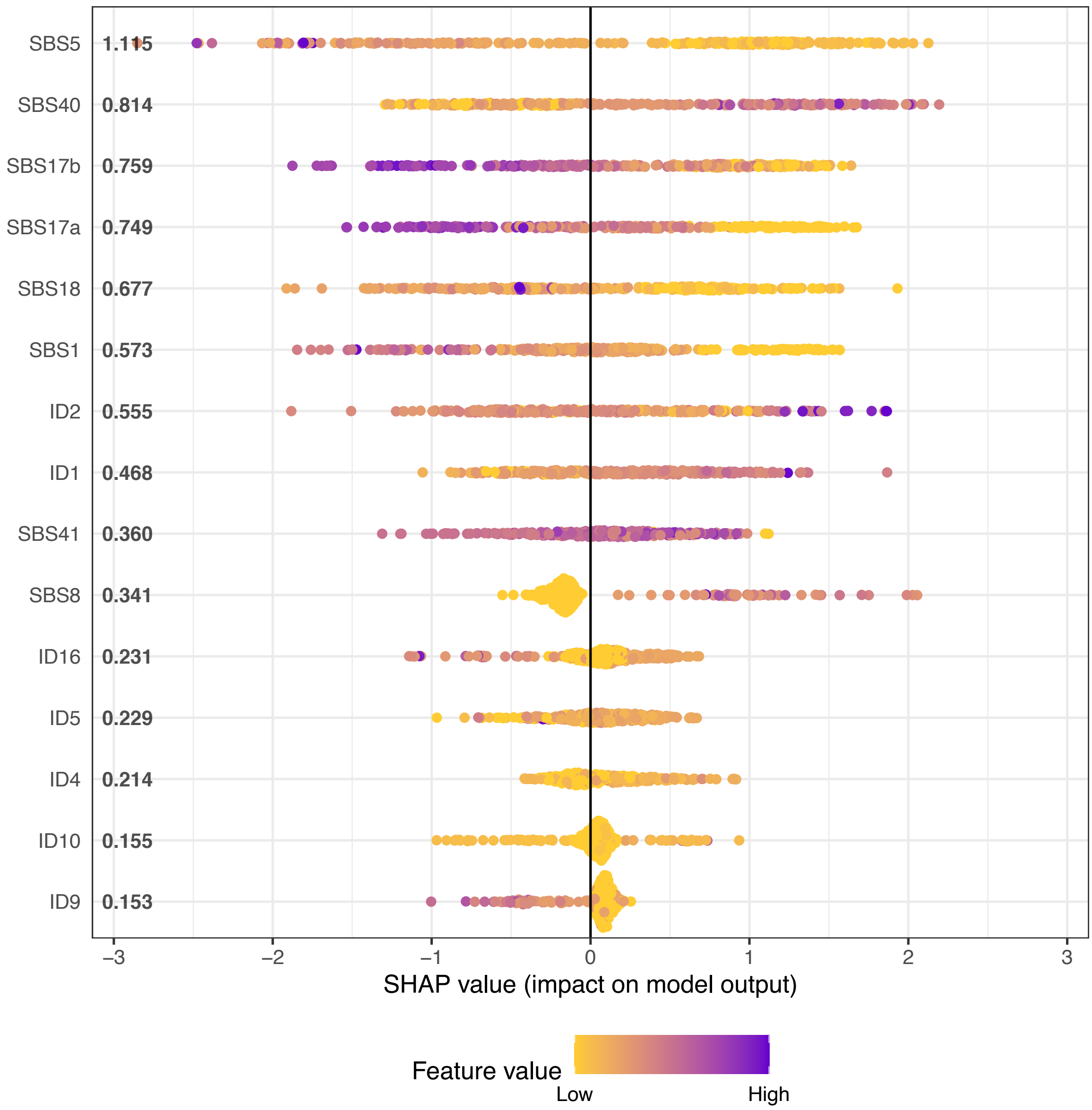

Supplementary Figure 24. Gradient boost classifier results distinguishing Barrett Oesophagus from primary tumours based on mutational and indel signature prevalence. Features are ordered according to their ranking in the model (top ranking features first). Every dot is a sample and the colour corresponds to the signature contribution in that sample, ranging from purple (highest contribution of the respective signature across the cohort) to yellow (lowest contribution of the respective signature). Features linked with primary tumours have a positive Shapley score, those linked with Barrett Oesophagus a negative score. Source data are provided as a Source Data file.

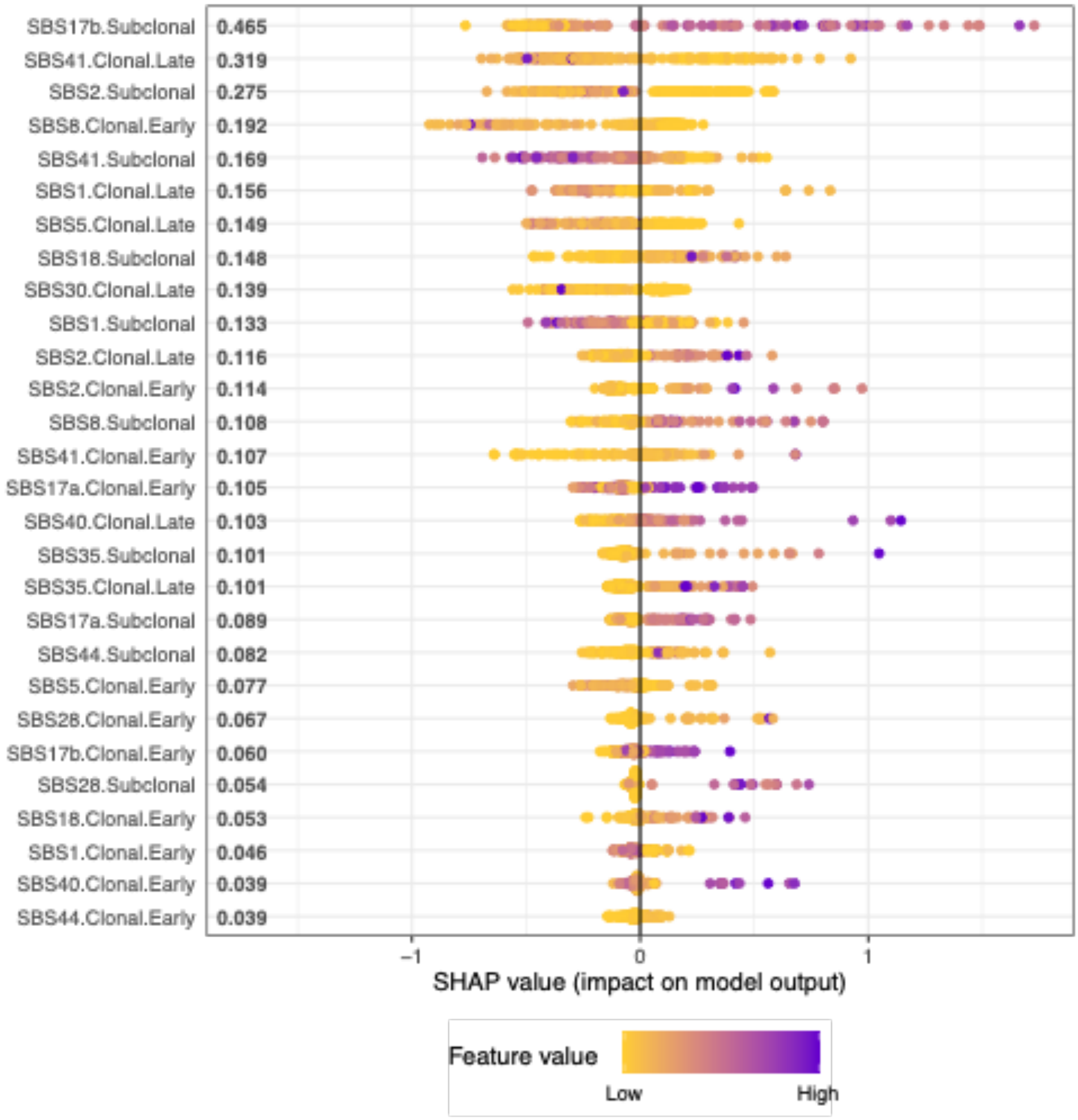

Supplementary Figure 25. Gradient boost classifier results distinguishing metastases from primary tumours based on mutational signature prevalence, clonality and timing. Features are ordered according to their ranking in the model (top ranking features first). Every dot is a sample and the colour corresponds to the signature contribution in that sample, ranging from purple (highest contribution of the respective signature across the cohort) to yellow (lowest contribution of the respective signature). Features linked with metastasis have a positive Shapley score, those linked with primary tumours a negative score. Source data are provided as a Source Data file.

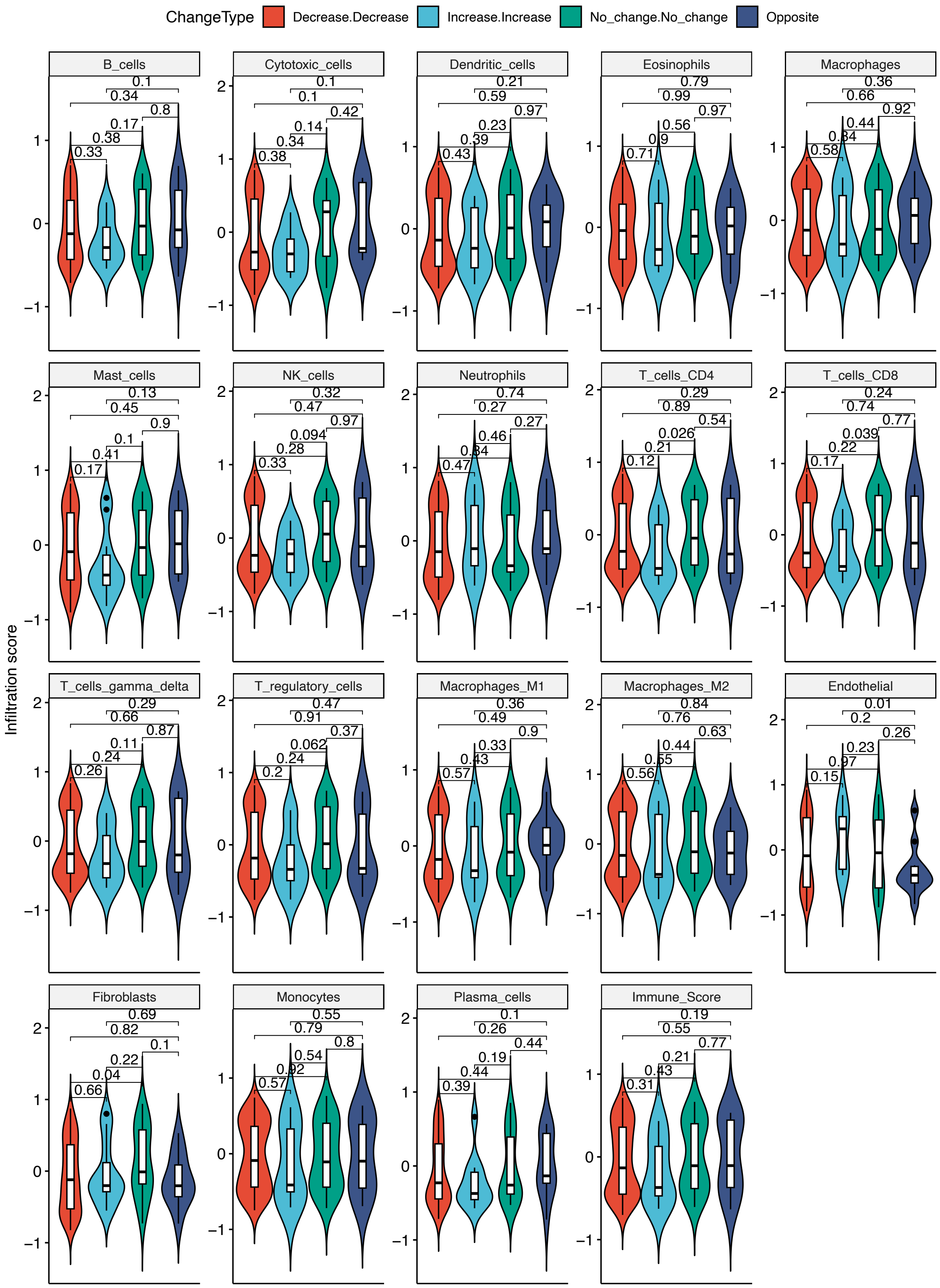

Supplementary Figure 26. Links between signature 17 dynamics and tumour microenvironment composition. Differences in non-tumour cell abundance between samples with different S17a/b subclonal dynamics are shown. Biologically independent

samples are compared, displaying: joint decrease in SBS17a and SBS17b (Decrease.Decrease, n=154), joint increase in SBS17a and SBS17b (Increase.Increase, n=11), no subclonal changes in either SBS17a or SBS17b (No_change.No_change, n=27), or opposite subclonal changes in SBS17a and SBS17b (Opposite, n=11). Box boundaries represent first and third quartiles, centerline indicates median values. The upper and lower whiskers extend from the hinges to the largest and smallest values, respectively, no further than 1.5 * the inter-quartile range. Two-sided Wilcoxon signed-rank test p-values are displayed. A joint subclonal increase in both SBS17a and SBS17b is linked with decreased infiltration of CD8+/CD4+ T cells, while a joint decrease in the signatures is accompanied by slight decrease in fibroblast abundance, as inferred by ConsensusTME. Source data are provided as a Source Data file.

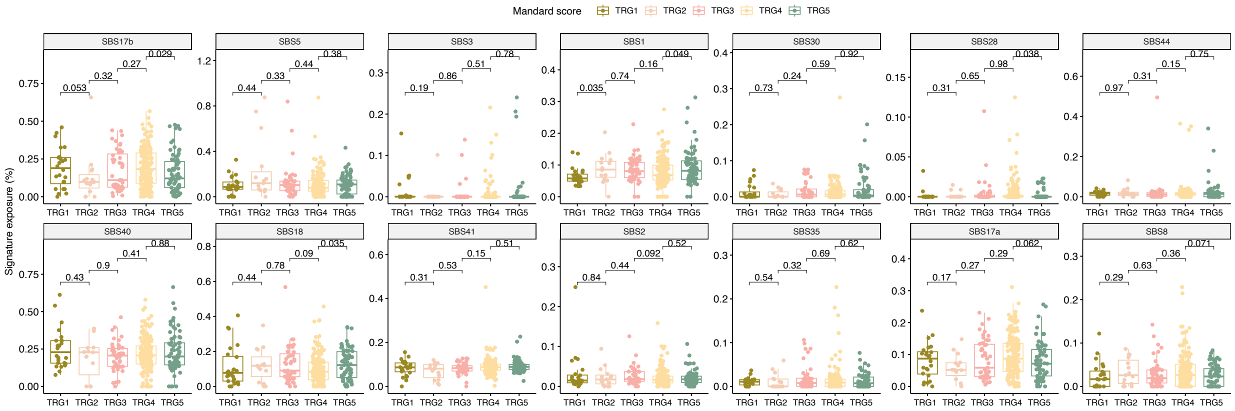

Supplementary Figure 27. Mutational signature prevalence by Mandard score. Mutational signature contributions are compared across pre/post-therapy samples belonging to different Mandard categories (n=21 TRG1, n=15 TRG2, n=47 TRG3, n=160 TRG4, n=73 TRG5 biologically independent samples). Box boundaries represent first and third quartiles, centerline indicates median values. The upper and lower whiskers extend from the hinges to the largest and smallest values, respectively, no further than 1.5 * the inter-quartile range. Two-sided Wilcoxon signed-rank test p-values are displayed. Source data are provided as a Source Data file.

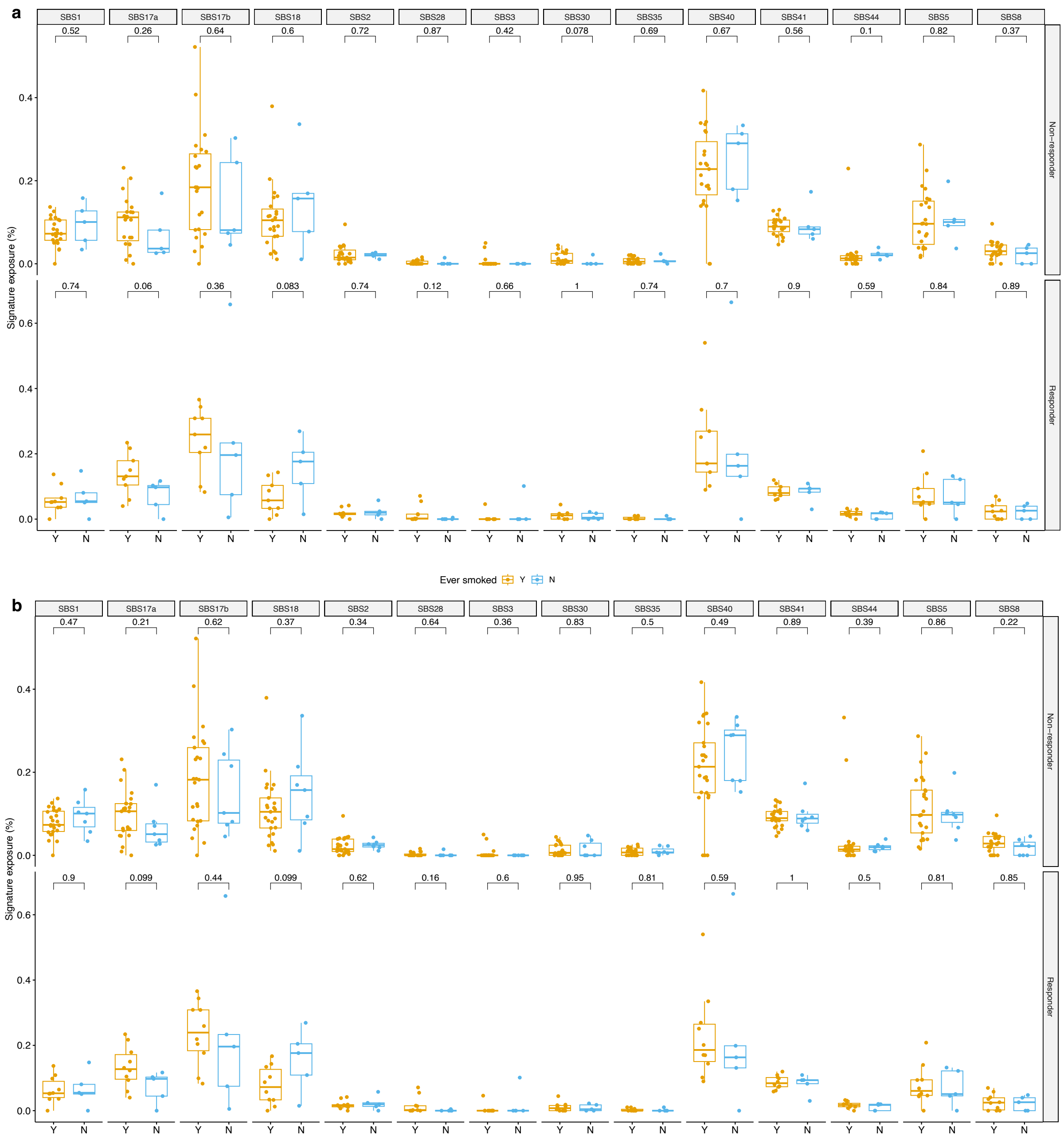

Supplementary Figure 28. Mutational signature exposures in pre-treatment tumours, compared between smokers and never smokers and split by treatment response. Mutational signature contributions in pre-treatment primary tumours are compared between past/present smokers (Y) and never smokers (N) within (a) responders (n=14) versus non-responders (n=26) to neoadjuvant chemotherapy and (b) responders (n=15) versus non-responders (n=30) to radiotherapy. Responders are defined as patients presenting complete or partial response; non-responders are patients with stable or progressive disease after treatment. Box boundaries represent first and third quartiles, centerline indicates median values. The upper and lower whiskers extend from the hinges to the largest and smallest values, respectively, no further than 1.5 * the inter-quartile range. Two-sided Wilcoxon signed-rank test p-values are displayed. Source data are provided as a Source Data file.

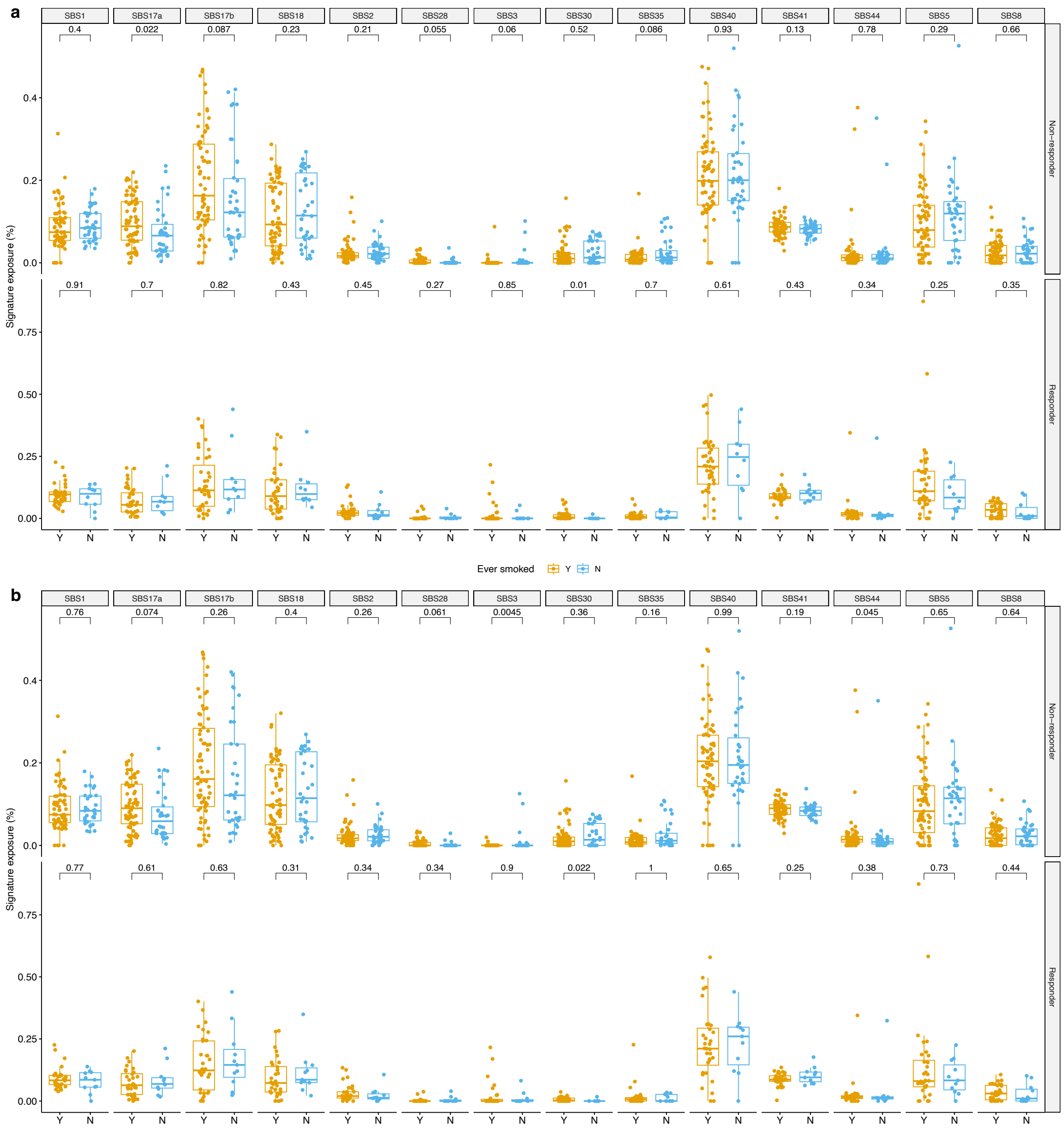

Supplementary Figure 29. Mutational signature exposures in post-treatment tumours, compared between smokers and never smokers and split by treatment response. Mutational signature contributions in post-treatment primary tumours are compared between past/present smokers (Y) and never smokers (N) within (a) responders (n=49) versus non-responders (n=97) to neoadjuvant chemotherapy and (b) responders (n=43) versus non-responders (n=92) to radiotherapy. Responders are defined as patients presenting complete or partial response; non-responders are patients with stable or progressive disease after treatment. Box boundaries represent first and third quartiles, centerline indicates median values. The upper and lower whiskers extend from the hinges to the largest and smallest values, respectively, no further than 1.5 * the inter-quartile range. Two-sided Wilcoxon signed-rank test p-values are displayed. Source data are provided as a Source Data file.

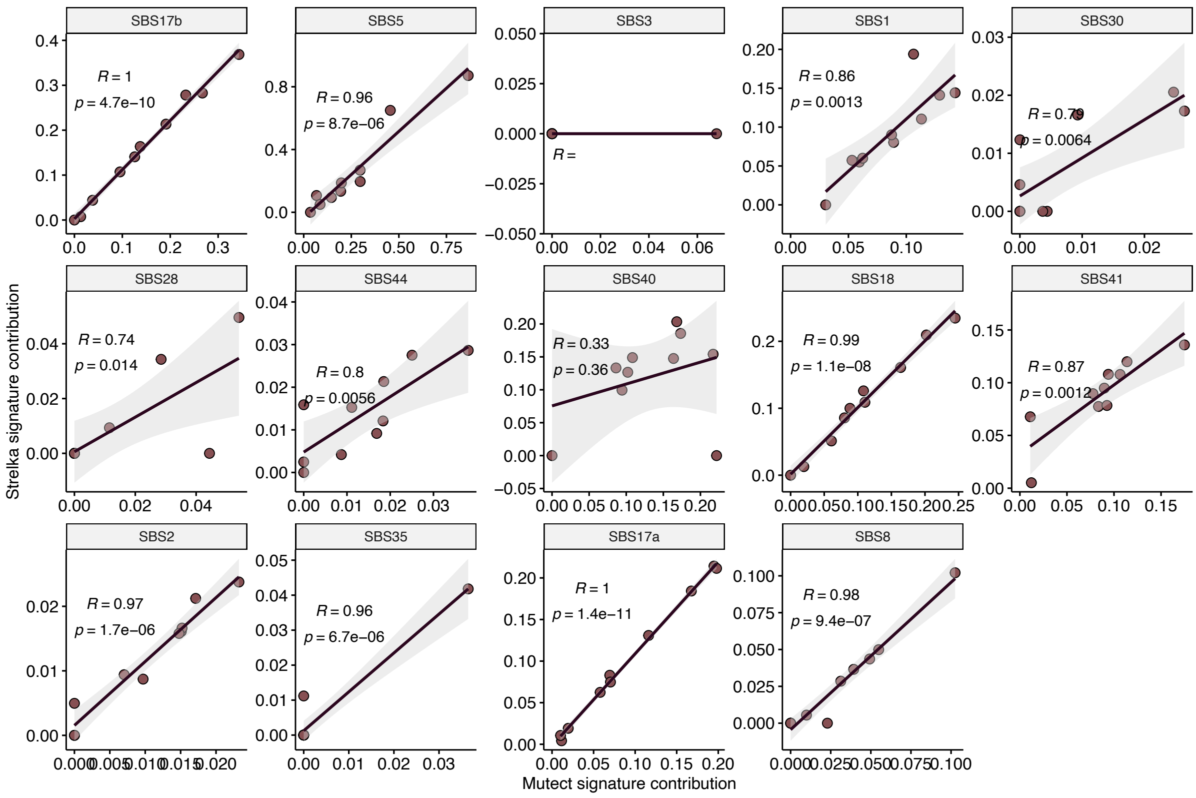

Supplementary Figure 30. Correlation in mutational signature exposure estimates between Strelka and MuTect2. Every dot corresponds to a sample. The two-sided Pearson correlation coefficient R and p-values between the mutational signature prevalence estimated from MuTect2 calls (x-axis) versus Strelka calls (y-axis) are displayed for each SBS signature. The shaded areas depict the 95% confidence intervals.

Source data are provided as a Source Data file.
