# Supplementary material for: Mutational signature dynamics shaping the evolution of oesophageal adenocarcinoma
Source: Nat Commun. 2023 Jul 15;14:4239. doi: 10.1038/s41467-023-39957-6 (PMC10349863; doi:10.1038/s41467-023-39957-6)
Supplement: Supplementary file 3 — Reporting Summary [file 41467_2023_39957_MOESM3_ESM.pdf]

Corresponding author(s): Rebecca C. Fitzgerald, Maria Secrier

Last updated by author(s): Jun 30, 2023

Reporting Summary

Nature Portfolio wishes to improve the reproducibility of the work that we publish. This form provides structure for consistency and transparency in reporting. For further information on Nature Portfolio policies, see our Editorial Policies and the Editorial Policy Checklist.

Statistics

For all statistical analyses, confirm that the following items are present in the figure legend, table legend, main text, or Methods section.

n/a	Confirmed	
☐	☒	The exact sample size (*n*) for each experimental group/condition, given as a discrete number and unit of measurement
☐	☒	A statement on whether measurements were taken from distinct samples or whether the same sample was measured repeatedly
☐	☒	The statistical test(s) used AND whether they are one- or two-sided<br>*Only common tests should be described solely by name; describe more complex techniques in the Methods section.*
☐	☒	A description of all covariates tested
☐	☒	A description of any assumptions or corrections, such as tests of normality and adjustment for multiple comparisons
☐	☒	A full description of the statistical parameters including central tendency (e.g. means) or other basic estimates (e.g. regression coefficient) AND variation (e.g. standard deviation) or associated estimates of uncertainty (e.g. confidence intervals)
☐	☒	For null hypothesis testing, the test statistic (e.g. *F*, *t*, *r*) with confidence intervals, effect sizes, degrees of freedom and *P* value noted<br>*Give P values as exact values whenever suitable.*
☒	☐	For Bayesian analysis, information on the choice of priors and Markov chain Monte Carlo settings
☒	☐	For hierarchical and complex designs, identification of the appropriate level for tests and full reporting of outcomes
☐	☒	Estimates of effect sizes (e.g. Cohen's *d*, Pearson's *r*), indicating how they were calculated

Our web collection on statistics for biologists contains articles on many of the points above.

Software and code

Policy information about availability of computer code

Data collection	The data were collected in house and analysed in R version 4.1.2.
Data analysis	Tools employed:<br>FastQC (http://www.bioinformatics.babraham.ac.uk/projects/fastqc) v0.11.6<br>BWA-MEM 2.0<br>GATK 3.2-2<br>Strelka 2.0.15<br>Mutect2 v4.1.7.0<br>Variant Effect Predictor 92.0<br>ascatNgs v2.1<br>Trim Galore v0.6.1<br>STAR 2.7.9a<br>SigProfilerExtractor v1.1.4<br>Sigminer v2.2.0<br>desconstructSigs v1.9.0<br>MutationalPatterns v3.10.0<br>MutationTimer v1.00.0<br>TrackSig<br>dNdScv v0.1.0<br>xgboost R package v1.7.3.1<br>randomForest R package v4.7-1.1

glmnet R package v4.1-7
GenomicRanges R package v1.46.1
ConsensusTME R package v0.0.1.9000
survminer R package v0.4.9
ggpubr R package v0.6.0
ggplot R package v2_3.4.1

The scripts developed during the analysis presented here are available at: https://github.com/secrierlab/Mutational-Signatures-OAC.

For manuscripts utilizing custom algorithms or software that are central to the research but not yet described in published literature, software must be made available to editors and reviewers. We strongly encourage code deposition in a community repository (e.g. GitHub). See the Nature Portfolio guidelines for submitting code & software for further information.

Data

Policy information about availability of data

All manuscripts must include a data availability statement. This statement should provide the following information, where applicable:

- Accession codes, unique identifiers, or web links for publicly available datasets
- A description of any restrictions on data availability
- For clinical datasets or third party data, please ensure that the statement adheres to our policy

The raw DNA sequencing data used in this study have all been previously published and are deposited at the European Genome-Phenome Archive (EGA) under accession codes: EGAD00001007785 [https://ega-archive.org/datasets/EGAD00001007785] (whole-genome sequencing of primary tumours and matched normal), EGAD00001006083 [https://ega-archive.org/datasets/EGAD00001006083] (whole-genome sequencing of primary tumours and matched normals), EGAD00001005434 [https://ega-archive.org/datasets/EGAD00001005434] (whole-genome sequencing of primary tumours, Barrett Oesophagus, metastases and matched normals), EGAD00001006349 [https://ega-archive.org/datasets/EGAD00001006349] (whole-genome sequencing of Barrett Oesophagus samples and matched normals). The raw sequencing data are available under restricted access due to data privacy laws; access can be requested to the ICGC Data Access Compliance Office as described here: https://docs.icgc-argo.org/docs/data-access/daco/applying. The processed mutation data for 409 primary tumours employed in this study are also available at the ICGC Data Portal (https://dcc.icgc.org/), under accession code ESAD-UK [https://dcc.icgc.org/projects/ESAD-UK]. The GRCh38/hg38 patch release 13 of the human reference genome [https://www.ncbi.nlm.nih.gov/assembly/GCF_000001405.39/] has been employed in this study. Source data are provided with this paper.

Research involving human participants, their data, or biological material

Policy information about studies with human participants or human data. See also policy information about sex, gender (identity/presentation), and sexual orientation and race, ethnicity and racism.

Reporting on sex and gender	The assembled cohort comprises 85 female and 560 male patients with OAC, and 26 female and 121 male patients with Barrett Oesophagus, based on self-report. All results presented come from amalgamating human data from both sexes. Sex and gender have not been considered in the design of this study, because OAC has a high male dominance and thus any study looking at differences between male and female cancers would likely be underpowered given the available data. No filtering of the human data was performed based on sex or gender, but we do report this information in Supplementary Table 1 and account for this variable when modelling clinical outcomes.
Reporting on race, ethnicity, or other socially relevant groupings	Patient age did not differ significantly between Barrett Oesophagus and OAC cases (median of 67 versus 68, see Supplementary Table 1). Race, ethnicity or other socially relevant groupings are not annotated and have not been considered in this study.
Population characteristics	The population characteristics are presented in Supplementary Table 1.
Recruitment	A cohort was assembled comprising 161 Barrett, 777 OACs and 59 metastatic samples that had been collected through a multicentre UK wide study called OCCAMS (Oesophageal Cancer Classification And Molecular Stratification) and undergone whole genome sequencing (WGS) as part of the ICGC-International Cancer Genome Consortium. These included 47 pairs of matched Barrett Oesophagus and primary tumours from the same individuals, and four trios of matched Barrett Oesophagus, OAC and metastases. Part of the OAC tumours (214/777) were collected from Mutographs study with available clinical annotations. A sample from the Barrett/tumour/metastatic sample and a matched germline reference, which was ideally matched blood or if not available normal squamous oesophagus as far away from the tumour as possible (at least 5cm), was collected during surgical resection or by an endoscopic biopsy. All samples were snap-frozen.
Ethics oversight	The research performed in this study complies with all relevant ethical regulations. The study was approved by the Cambridge South Research Ethics Committee (REC 07/H0305/52 and 10/H0305/1) and included written individual informed consent. No participant compensation was provided.

Note that full information on the approval of the study protocol must also be provided in the manuscript.

Field-specific reporting

Please select the one below that is the best fit for your research. If you are not sure, read the appropriate sections before making your selection.

☒ Life sciences ☐ Behavioural & social sciences ☐ Ecological, evolutionary & environmental sciences

For a reference copy of the document with all sections, see nature.com/documents/nr-reporting-summary-flat.pdf

Life sciences study design

All studies must disclose on these points even when the disclosure is negative.

Sample size	All samples with available DNA sequencing data that have been collected through OCCAMS have been employed in this study, with no pre-calculation of the sample size. With 161 Barrett Oesophagus samples, 777 primary tumours and 59 metastases, the study is well powered to uncover changes in mutational processes across disease stages.
Data exclusions	A systematic pathological review was performed to check the cellularity of the tumour samples using hematoxylin-and eosin-stained sections and only samples with >70% cellularity were included.
Replication	The analytical procedures implemented here are highly reproducible, consisting of fixed steps and parameters, and the code has been made freely available at the following repository: https://github.com/secrierlab/Mutational-Signatures-OAC.<br>When modelling of disease stages based on mutational signature prevalence, we employed two machine learning procedures (gradient boost and random forest classifiers) to ensure the robustness of the analysis.
Randomization	Samples were split into groups according to the respective disease stage (Barrett Oesophagus, primary tumour, metastasis). The analysis did not involve any random allocation of samples.
Blinding	Blinding was not applied to this study because knowing the stage of the disease (Barrett Oesophagus, primary tumour, metastasis) was integral to the analysis.

Reporting for specific materials, systems and methods

We require information from authors about some types of materials, experimental systems and methods used in many studies. Here, indicate whether each material, system or method listed is relevant to your study. If you are not sure if a list item applies to your research, read the appropriate section before selecting a response.

Materials & experimental systems

n/a	Involved in the study	
☒	☐	Antibodies
☒	☐	Eukaryotic cell lines
☒	☐	Palaeontology and archaeology
☒	☐	Animals and other organisms
☒	☐	Clinical data
☒	☐	Dual use research of concern
☒	☐	Plants

Methods

n/a	Involved in the study	
☒	☐	ChIP-seq
☒	☐	Flow cytometry
☒	☐	MRI-based neuroimaging
